# Activation of the ATF2/CREB-PGC-1α pathway by metformin leads to dopaminergic neuroprotection

**DOI:** 10.18632/oncotarget.18122

**Published:** 2017-05-24

**Authors:** Hojin Kang, Rin Khang, Sangwoo Ham, Ga Ram Jeong, Hyojung Kim, Minkyung Jo, Byoung Dae Lee, Yun Il Lee, Areum Jo, ChiHu Park, Hyein Kim, Jeongkon Seo, Sun Ha Paek, Yun-Song Lee, Jeong-Yun Choi, Yunjong Lee, Joo-Ho Shin

**Affiliations:** ^1^ Department of Molecular Cell Biology, Division of Pharmacology, Sungkyunkwan University School of Medicine, Samsung Biomedical Research Institute, Suwon, South Korea; ^2^ Single Cell Network Research Center, Sungkyunkwan University School of Medicine, Suwon, South Korea; ^3^ Department of Neuroscience, Department of Physiology, Neurodegeneration Control Research Center, Kyung Hee University School of Medicine, Seoul, South Korea; ^4^ Department of New Biology, Daegu Geongbuk Institute of Science and Technology, Daegu, South Korea; ^5^ Research Core Facility, Sungkyunkwan University School of Medicine, Samsung Biomedical Research Institute, Suwon, South Korea; ^6^ HuGeX Co., Ltd. Seongnam, South Korea; ^7^ UNIST Central Research Facility, Ulsan National Institute of Science and Technology, Ulsan, South Korea; ^8^ Department of Neurosurgery, Seoul National University College of Medicine, Seoul, South Korea

**Keywords:** metformin, dopaminergic, Parkinson's disease, PGC-1α, mitochondria, Gerotarget

## Abstract

Progressive dopaminergic neurodegeneration is responsible for the canonical motor deficits in Parkinson's disease (PD). The widely prescribed anti-diabetic medicine metformin is effective in preventing neurodegeneration in animal models; however, despite the significant potential of metformin for treating PD, the therapeutic effects and molecular mechanisms underlying dopaminergic neuroprotection by metformin are largely unknown.

In this study, we found that metformin induced substantial proteomic changes, especially in metabolic and mitochondrial pathways in the substantia nigra (SN). Consistent with this data, metformin increased mitochondrial marker proteins in SH-SY5Y neuroblastoma cells. Mitochondrial protein expression by metformin was found to be brain region specific, with metformin increasing mitochondrial proteins in the SN and the striatum, but not the cortex. As a potential upstream regulator of mitochondria gene transcription by metformin, PGC-1α promoter activity was stimulated by metformin via CREB and ATF2 pathways. PGC-1α and phosphorylation of ATF2 and CREB by metformin were selectively increased in the SN and the striatum, but not the cortex. Finally, we showed that metformin protected dopaminergic neurons and improved dopamine-sensitive motor performance in an MPTP-induced PD animal model. Together these results suggest that the metformin-ATF2/CREB-PGC-1α pathway might be promising therapeutic target for PD.

## INTRODUCTION

Progressive loss of dopaminergic neuron and accountable motor deficits characterize Parkinson's disease (PD), the most common neurodegenerative movement disorder [1, 2]. Although treatment with L-DOPA alleviates motor dysfunction in PD patients, long-term treatment can result in adverse effects such as dyskinesia [2, 3]. Other than the symptomatic relief provided by L-DOPA treatment or deep brain stimulation, there are no proven therapies capable of preventing the progressive loss of dopaminergic neurons in PD [3, 4]. Therefore, it is important to develop new therapeutic strategies based upon the molecular pathogenesis of PD.

Most cases of PD are considered sporadic, although several genes have been identified in inherited cases of PD [2]. Genetic and functional studies on PD-causing gene mutations (i.e., *a-synuclein, Parkin, PINK1, LRRK2*, and *DJ-1*) have provided insight into the diverse and varied molecular mechanisms by which dopaminergic dysfunction and degeneration occur [5]. Previous reviews on the molecular pathophysiology of PD [5, 6] indicate that PD genes interact with mitochondria to ultimately cause oxidative stress and cell toxicity. Mitochondrial toxins such as 1-methyl-4-phenyl-1,2,3,6-tetrahydropyridine (MPTP) and rotenone have been well characterized for their ability to recapitulate major PD-related pathologies including mitochondrial dysfunction, oxidative stress, and dopaminergic cell loss in animal models of PD [7–9]. Interestingly, protective PD genes such as *Parkin* and *PTEN-induced putative kinase 1 (PINK1)* [10, 11] protect against these mitochondrial toxins, whereas autosomal dominant mutations of *a-SYNUCLEIN* or *Leucine-rich repeat kinase 2 (LRRK2)* exacerbate the toxicities elicited by mitochondrial insults [12, 13]. Thus, mitochondrial dysfunction appears to be involved in major PD-related pathologies.

Consistent with the role of mitochondrial dysfunction in the pathogenesis of PD, numerous studies have pointed to the role of peroxisome proliferator-activated receptor gamma coactivator 1-alpha (PGC-1α) in dopaminergic neuronal survival in PD [14–18]. PGC-1α is a transcriptional cofactor and master regulator of mitochondrial biogenesis and anti-oxidant defense [19, 20]. A genome-wide meta-analysis revealed that various PGC-1α target genes are downregulated in PD patient brains [15], suggesting that dysfunctional PGC-1α is associated with the clinical pathogenesis of PD. In addition, *PGC-1α* knockout mice are more vulnerable to dopaminergic neurodegeneration caused by the mitochondrial toxin MPTP [21]. Conditional *Parkin* ablation in mice leads to PGC-1α repression via the accumulation of the Parkin-interacting substrate, ZNF746 (PARIS) in dopaminergic neurons [14]. Conversely, PGC-1α overexpression protects against dopaminergic neurodegeneration in PD mouse models [17]. As such, strategies to boost mitochondrial structure and function through modulating PGC-1α could be beneficial in preventing dopaminergic cell loss in PD.

Metformin is a widely prescribed anti-diabetic drug that normalizes disturbed homeostasis of glucose metabolism in type II diabetes patients. Metformin's glucose-lowering effects are mediated by its diverse cellular effects on liver, skeletal muscle, and fat tissues [22]. Several diverse molecular mechanisms of metformin have been identified [22], and it appears that stimulation of AMP-activated protein kinase (AMPK) by metformin mediates its beneficial effect on diabetes [22, 23]. Since metabolic deregulation is accountable for neurodegeneration in several brain disorders, metformin has been reported to be neuroprotective in many diseases including stroke [24], Huntington's disease [25], Alzheimer's disease [26], and PD [27]. Since these neurodegenerative diseases involve diverse brain regions and neuronal subtypes, it is largely unknown which molecular mechanisms underlie the global neuroprotective effects of metformin. Especially, metformin's effects on the survival of dopaminergic neurons in PD mouse models are controversial. Although several reports have demonstrated a beneficial effect of metformin, AMPK activation by metformin in 6-hydroxydopamine (6-OHDA)-intoxicated dopaminergic neurons exacerbates progressive dopaminergic cell loss [28, 29].

In the present study, we found that metformin primarily alters metabolic and mitochondrial pathways in the substantia nigra (SN) of mouse brains by upregulating mitochondrial protein expression. In addition, SN-specific PGC-1α induction by metformin was found to contribute to mitochondrial biogenesis. Finally, we showed that metformin induces PGC-1α via Activating Transcription Factor 2 (ATF2)/ cAMP response element binding protein (CREB) signaling, which is critical for the neuroprotective effects of metformin. This study suggests novel molecular mechanism by which metformin facilitates dopaminergic neuroprotective effects against mitochondrial insults and suggests the potential application of metformin in the maintenance of dopaminergic neuronal function.

## RESULTS

### Metformin alters metabolic and mitochondrial pathways in the mouse SN

**Figure 1:**
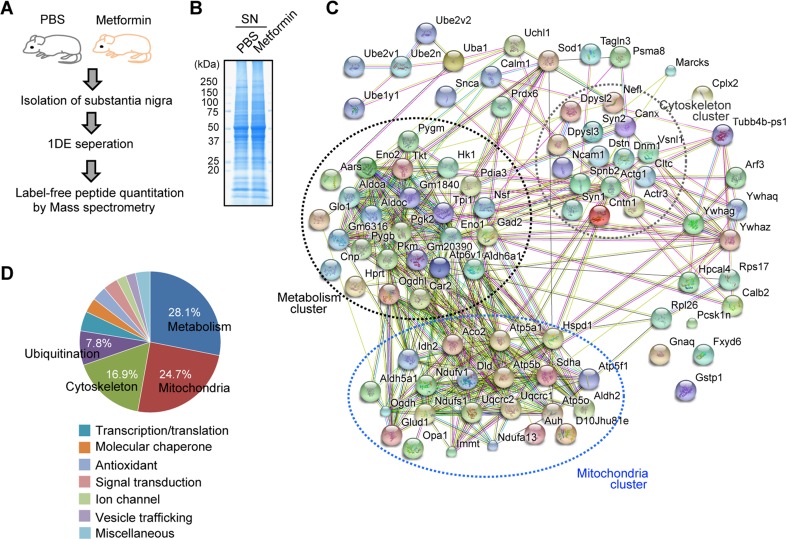
Metformin induces global proteomic alterations in the mouse SN **A**. Experimental scheme for metformin treatment and mass spectrometry. **B**. Representative 1DE image of substantia nigra (SN) proteins of PBS- or metformin-administrated mice by Coommassie staining. **C**. Proteins with differential expression between PBS and metformin treatment were analyzed by functional clustering. Biological pathway clusters enriched in the proteome of differential expression are indicated. Line color annotation: green, neighborhood; red, gene fusion; blue, co-occurrence; black, coexpression; pink, experiments; light blue, databases; light green, text mining; light blue, homology. **D**. Phi chart showing biological pathways enriched with multiple genes differentially expressed between the PBS and metformin treatment groups. The total number of genes with differential expression in each pathway were normalized and expressed as percentages.

To obtain insight into how metformin affects the brain and mediates potential neuroprotective effects in PD on a global level, we performed label-free proteomics using tissue from the SN, which contains a significant population of dopaminergic neurons (Figure 1). Frontal cortex was also included for comparison with SN (Supplementary Figure 1). Mice were treated with metformin or PBS via drinking water for 2 weeks. The SN and cortex were then dissected, and total proteins were separated by SDS-PAGE electrophoresis (Figure 1B and Supplementary Figure 1A). Protein identification and relative quantification were performed with mass spectrometry and label-free peptide quantification. Proteins with altered expression in the SN of metformin-treated mice were grouped into functional pathways and visualized (Figure 1C). We found that metformin-induced proteins were highly enriched in several distinct pathways including metabolism (28%), mitochondria (25%), cytoskeleton (17%), and ubiquitination (8%) (Figures 1C and 1D, Supplementary Table 1). Interestingly, cortex proteomic alteration by metformin was different from SN proteomic changes. Comparative proteomics revealed that signal transduction (21%) and metabolism (14%) proteins were mainly altered in the cortex of metformin-administrated mice. Moreover, there were fewer protein functional network clusters as compared to SN protein network (Supplementary Figure 1B and Supplementary Figure 1C, Supplementary Table 2). Overall, global proteomic analysis revealed that metformin has a major impact on metabolic and mitochondrial pathways in the SN, both of which are reciprocally regulated.

### Nigrostriatal region-specific increase of mitochondrial proteins by metformin

**Figure 2:**
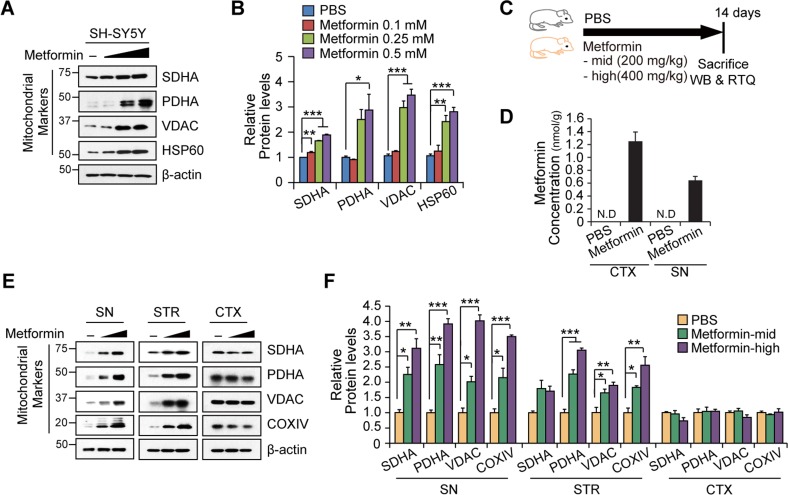
Nigrostriatal region-specific increase of mitochondrial proteins by metformin **A**. Representative Western blot of mitochondrial marker proteins (SDHA, PDHA, VDAC, and HSP60) in total protein lysates from SH-SY5Y cells treated with different doses (0.1, 0.25, 0.5 mM) of metformin for 48hr. b-actin was used as an internal loading control. **B**. Quantification of relative mitochondrial protein levels in SH-SY5Y cells according to treatment and normalized to -actin (*n* = 3 per group). **C**. Cartoon of experimental plan. Drinking water containing PBS or metformin was supplied for 14 days. **D**. Mass spectrometric analysis confirmed brain-penetrated metformin (*n* = 3 per group). **E**. Representative Western blots of mitochondrial marker proteins in the indicated brain subregions (SN, STR, and CTX) from mice treated with PBS or different doses of metformin for 7 days. b-actin was used as an internal loading control. **F**. Quantification of relative mitochondrial protein levels in brain subregions from the indicated experimental mouse groups. Band intensities of each protein were normalized to b-actin and compared to the PBS group (*n* = 3 per group). Quantified data are expressed as the mean ± SEM. Statistical significance was determined by unpaired two-tailed Student's *t* test or ANOVA test with Tukey post-hoc analysis, *p < 0.05, **p< 0.01 and ***p < 0.001.

To further confirm the mitochondrial impact of metformin treatment, we treated the SH-SY5Y dopaminergic neuroblastoma cell line with increasing doses of metformin and monitored the expression of mitochondrial marker proteins. The mitochondrial marker proteins that were tested included the succinate dehydrogenase complex, subunit A (SDHA), pyruvate dehydrogenase (PDHA), voltage-dependent anion channel (VDAC), and heat shock protein 60 (HSP60). All of these proteins were upregulated in SH-SY5Y cells by metformin in a dose-dependent manner (Figures 2A and 2B). We next examined whether different doses of metformin regulate mitochondrial proteins *in vivo* (Figure 2C). To this end, mice were given two different doses of metformin (Mid: 200 mg/kg and High: 400 mg/kg) for 14 days via drinking water. Successful delivery of metformin to SN and cortex was confirmed by mass spectrometry (Figure 2D). Three brain subregions (SN, STR; striatum, and CTX; cortex) were then analyzed by Western blot to monitor changes in the expression of mitochondrial marker proteins, including SDHA, PDHA, VDAC, and cytochrome C oxidase subunit IV (COXIV). Interestingly, there was a dose-dependent increase in all of the mitochondrial marker proteins in the SN and all but SDHA in the striatum tissues, whereas metformin failed to upregulate these marker proteins in the cortex (Figures 2E and 2F). These data suggest that metformin increases mitochondrial mass in SH-SY5Y cells, and that metformin's effect on mitochondria *in vivo* is notably restricted to the nigrostriatal regions.

### ATF2/CREB pathway activation by metformin mediates PGC-1α induction

**Figure 3:**
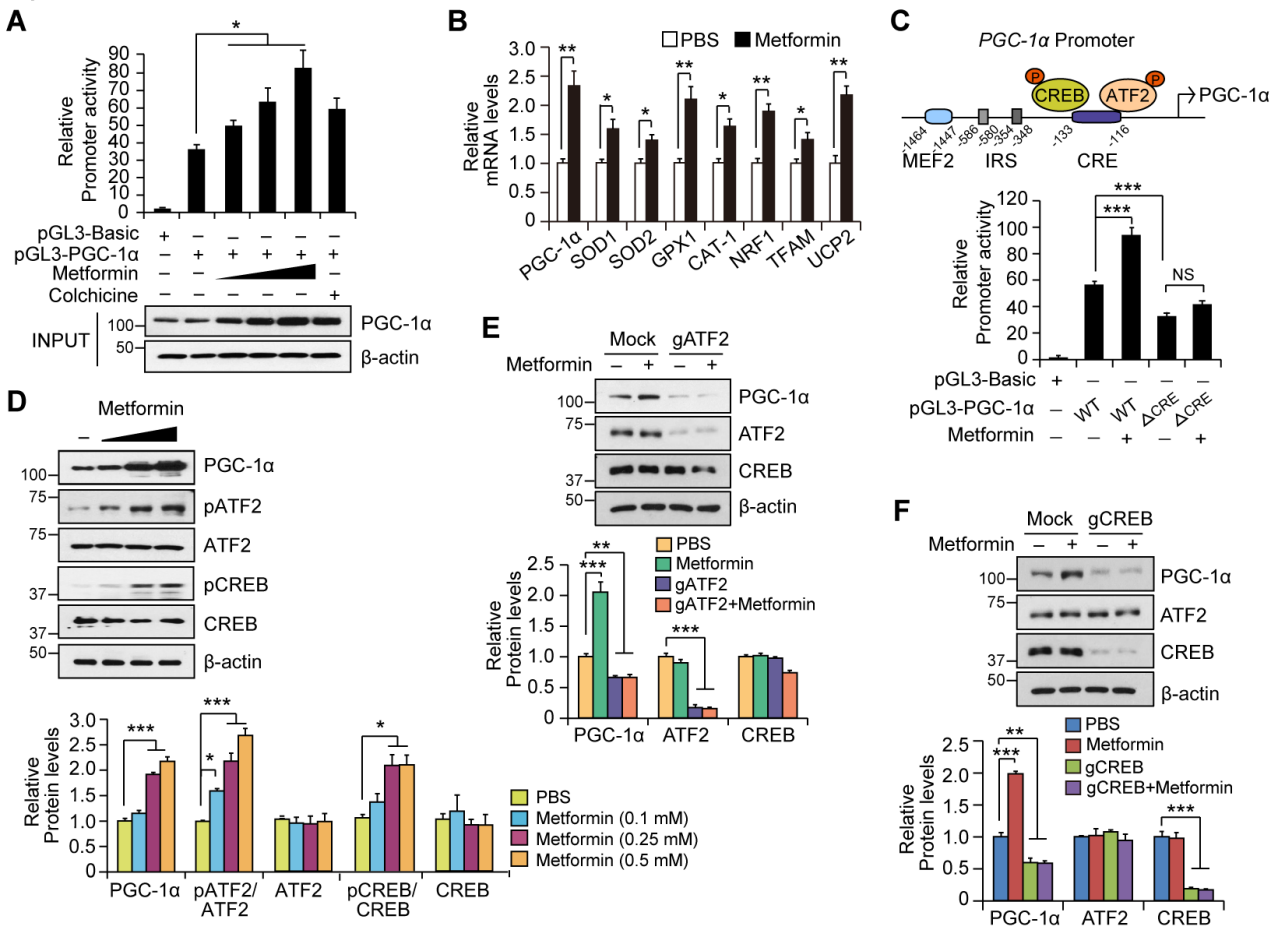
Metformin increases PGC-1α expression by activating the ATF2/CREB pathway **A**. Quantification of relative PGC-1 promoter activity in SH-SY5Y cells transiently transfected with a pGL3-PGC-1α luciferase construct and treated with metformin (0.1, 0.25, 0.5 mM), PBS, or positive control (colchicine) for 48hr. Promoter activity was determined by luciferase assay (*n* = 3 independent experiments). The bottom panel shows PGC-1α protein levels in each group determined by Western blot using an anti-PGC-1 antibody. b-actin was used as an internal loading control. **B**. Quantification of relative PGC-1α messenger and its target gene levels in SH-SY5Y cells treated with either PBS or 0.5 mM metformin for 48hr as determined by real-time quantitative PCR (*n* = 3 per group). The abundance of each gene was normalized to the ACTB as internal control. **C**. In the top panel, illustration of *PGC-1α* promoter was demonstrated. Quantification of relative PGC-1α promoter activity in SH-SY5Y cells transiently transfected with a luciferase construct with a PGC-1α wild type or CRE deletion mutant promoter. Promoter activity was measured by luciferase assay following metformin or PBS treatment for 48hr (bottom panel, *n* = 3 per group). **D**. Expression of PGC-1α and its upstream regulators (CREB and ATF2) in SH-SY5Y cells treated with either PBS or different doses of metformin (0.1, 0.25, 0.5 mM) for 48hr, as determined by Western blot with the indicated antibodies (upper panel). Quantification of PGC-1α and relative phosphorylation of CREB and ATF2 in SH-SY5Y cells treated with either PBS or metformin (lower panel). Phospho-CREB and phospho-ATF2 band intensities were normalized to total CREB and ATF2, respectively (*n* = 3 per group). **E**. PGC-1α induction by 0.5 mM metformin treatment for 48hr, in SH-SY5Y cells with ATF2 knockout by transient transfection of indicated gRNA constructs as determined by Western blot using the indicated antibodies (upper panel). Quantification of relative PGC-1α, ATF2, and CREB levels normalized to b-actin in each group is shown in the lower panel (*n* = 3 per group). **F**. PGC-1α induction by 0.5 mM metformin treatment for 48hr in SH-SY5Y cells with CREB knockout by transient transfection of indicated gRNA constructs determined by Western blots using the indicated antibodies (upper panel). Quantification of relative PGC-1α, ATF2, and CREB levels normalized to b-actin in each group is shown in the lower panel (*n* = 3 per group).

We next explored the potential upstream regulators that facilitate the increased mitochondrial protein expression by metformin. Initially, we hypothesized that metformin might activate PGC-1α, which is a master regulator of mitochondrial biogenesis and antioxidant defense [19, 20]. To evaluate this possibility, SH-SY5Y cells were transiently transfected with a *PGC-1α* promoter luciferase construct (pGL3-PGC-1α) and treated with metformin. Metformin increased *PGC-1α* promoter activity in a dose-dependent fashion (Figure 3A). Specifically, high-dose metformin increased *PGC-1α* promoter luciferase activity by approximately three-fold compared to vehicle, while colchicine (10 μM), a known inducer of PGC-1α and positive control, increased PGC-1α luciferase activity by almost two-fold. The results of the *PGC-1α* promoter luciferase assay were well correlated with actual PGC-1α protein expression and messenger levels as determined by Western blot and real-time qRT-PCR, respectively (Figures 3A and 3B). Next, we found that metformin increased mRNA levels of selected PGC-1α target genes, including superoxide dismutase-1 (*SOD1*), superoxide dismutase-2 (*SOD2*), glutathione peroxidase-1 (*GPX1*), cationic amino acid transporter-1 (*CAT1*), nuclear respiratory factor-1 (*NRF1*), mitochondrial transcription factor A (*TFAM*), and uncoupling protein-2 (*UCP2*) (Figure 3B).

The PGC-1α promoter is regulated by several transcriptional activators and repressors [20] (as shown in the upper panel of Figure 3C for the structures of *PGC-1α* promoter and its potential regulators). To narrow the potential mechanisms of PGC-1α induction by metformin, we examined whether metformin affects expression of the well-known PGC-1α repressor PARIS (the Parkin Interacting Substrate, ZNF 746). Metformin had no effect on the expression of PARIS in SH-SY5Y cells (Supplementary Figure 2A). Moreover, upregulation of PGC-1α by metformin did not appear to be PARKIN-dependent because human adipose tissue-derived mesenchymal stromal cells (hAD-MSCs) from human adult patients with early-onset hereditary familial *PARKIN*-mutations or late-onset idiopathic PD continued to display increased PGC-1α protein levels induced by metformin (Supplementary Figures 2B, 2C, and 2D). Metformin has been shown to regulate metabolic parameters via activation of AMPK in muscle and fat tissue [22, 30]. Treatment of SH-SY5Y cells with metformin failed to increase AMPK phosphorylation (Supplementary Figure 2E), indicating PGC-1α induction by metformin in this neuroblastoma cell line is AMPK independent.

CREB and ATF2 are transcriptional activators of PGC-1α that, when phosphorylated and dimerized, bind to cAMP response element (CRE) within the *PGC-1α* promoter [20]. To determine whether the CRE motif within the *PGC-1α* promoter is important for metformin-induced PGC-1α expression, we performed a luciferase assay in SH-SY5Y cells transiently transfected with a *PGC-1α* mutant luciferase construct in which the CRE motif was deleted *(Δ*CRE). The metformin-induced 70% enhancement of *PGC-1α* promoter activity was abolished in SH-SY5Y cells transfected with the *PGC-1α Δ*CRE luciferase construct (Figure 3C). Further, the luciferase activity of the PGC-1α ΔCRE promoter was decreased below the basal promoter activity of the wild type *PGC-1α* promoter (Figure 3C). Taken together, these results suggest that metformin increases PGC-1α expression through the CRE motif, and that this motif is important for maintenance of basal PGC-1α expression under physiological conditions.

We next analyzed whether CREB and ATF2 are activated following metformin treatment in SH-SY5Y cells. Treatment with metformin increased the phosphorylation status of CREB and ATF2 by two- to three-fold, as determined by immunoblot analysis, which correlated with PGC-1α induction (Figures 3D). To further determine the role of CREB or ATF2 in mediating metformin's effect on PGC-1α expression, we transiently transfected SH-SY5Y cells with a guide RNA (gRNA) targeting *CREB* or *ATF2* along with Cas9 expression to knockout each gene and subsequently examined the effects on PGC-1α expression by immunoblot analysis. The knockout strategy reduced the expression of both CREB and ATF2 by almost 80%. Following deletion of *CREB* or *ATF2* in SH-SY5Y cells, metformin no longer induced PGC-1α expression (Figures 3E and 3F), indicating that both genes are necessary for PGC-1α expression downstream of metformin treatment. Significantly, ablation of either *ATF2* or *CREB* in SH-SY5Y cells under physiological conditions led to an approximately 40% reduction in basal PGC-1α expression, which again suggests a role of ATF2 and CREB in maintaining basal PGC-1α expression.

Since CREB and ATF2 have been shown to be activated by environmental changes and cellular stress and, in turn, induce the expression of adaptive genes [31–33], we next determined if metformin elevates levels of oxidative stress in a dose-dependent manner in SH-SY5Y cells. Treatment of SH-SY5Y cells with 0.5 mM metformin led to an approximately 30% increase in ROS level (Supplementary Figure 3A), which may have contributed to CREB/ATF2 activation. Consistent with this notion, low concentrations of hydrogen peroxide, which also elevate ROS levels up to 30% of basal levels, increased relative phospho-CREB, total ATF2, and phospho-ATF2 levels in SH-SY5Y cells (Supplementary Figure 3B). In addition, low concentrations of hydrogen peroxide induced a concomitant increase in PGC-1α in SH-SY5Y cells (Supplementary Figure 3B). PGC-1α is an important regulator of mitochondrial biogenesis; thus, we also assessed ATP production as an indicator of metabolic parameters. Low concentration of hydrogen peroxide, at which there was PGC-1α induction, increased ATP levels by about 30% as compared to control (Supplementary Figure 3C). More potently, treatment with increasing doses of metformin elevated ATP content up to 80% in SH-SY5Y cells (Supplementary Figure 3C), and these concentrations of metformin increased PGC-1α expression by activating ATF2/CREB (Figure 3D).

### Metformin increases PGC-1α in the nigrostriatal regions in vivo

**Figure 4:**
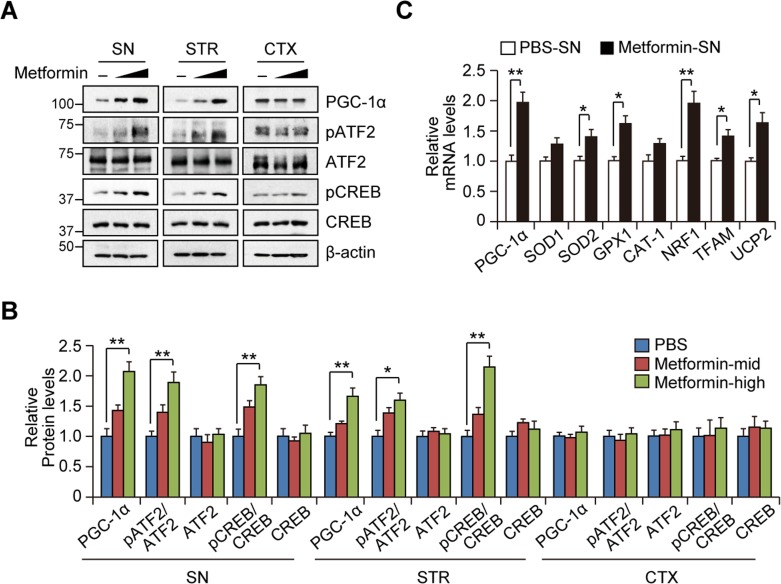
Region-specific induction of PGC-1α by metformin treatment *in vivo* **A**. Representative Western blots showing PGC-1α induction and CREB or ATF2 phosphorylation by metformin treatment for 14 days (Mid : 200 mg/kg/day, High : 400 mg/kg/day) in SN, STR, and CTX subregions. **B**. Quantification of relative PGC-1α, phospho-CREB, and phospho-ATF2 levels in the indicated brain regions of mice treated with either PBS or metformin (*n* = 3 per group). **C**. Quantification of relative PGC-1α and its target gene messenger levels in the SN of mice treated with either PBS or metformin (*n* = 3 per group).

We next aimed to extend our observations of PGC-1α regulation by metformin in SH-SY5Y cells to an *in vivo* mouse model. Western blot analysis in three brain subregions (SN, STR, and CTX) from mice treated with metformin revealed a dose-dependent increase of PGC-1α protein in the nigrostriatal regions including SN and STR (Figures 4A and 4B). We also observed increased CREB and ATF2 phosphorylation in the nigrostriatal regions, which correlated with PGC-1α expression and was consistent with our observations in SH-SY5Y cells. However, we did not observe any changes in PGC-1α or CREB/ATF2 phosphorylation in the cortex tissues following treatment with metformin (Figures 4A and 4B). We next determined whether the increase in PGC-1α expression by metformin in the SN was regulated at the level of transcription. Real-time quantitative RT-PCR showed the upregulation of PGC-1α and messenger levels of its target genes including SOD2, GPX1, NRF1, TFAM, and UCP2 in the SN from metformin-treated mice (Figure 4C).

### Metformin protects dopaminergic neurons in the MPTP mouse model

**Figure 5:**
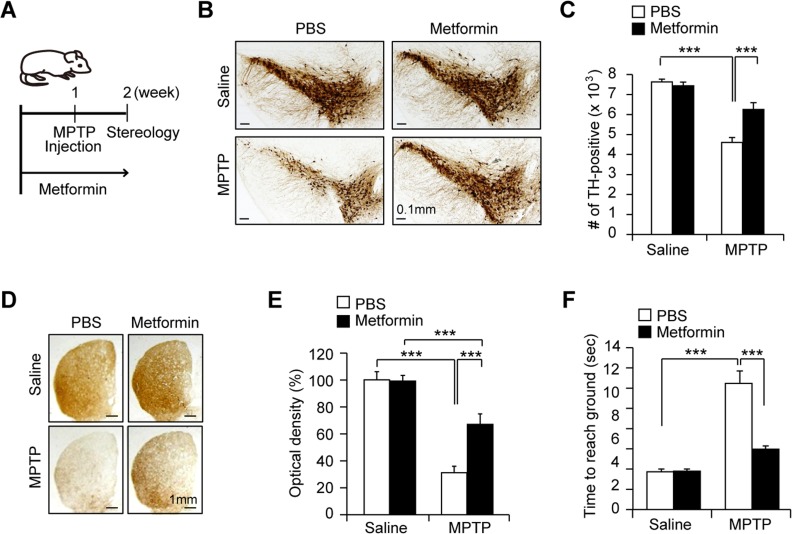
Dopamine neuron protection by metformin in the MPTP mouse model **A**. Scheme of experimental plan. After 7 days pretreatment of metformin via drinking water, MPTP was injected. Stereological analysis was followed post MPTP-injection 7 days. **B**. Representative image of immunohistochemistry using anti-TH antibodies for SN coronal brain sections containing major dopaminergic neuron clusters from the indicated mouse treatment groups. **C**. Stereological assessment of total TH-positive dopaminergic neurons in the SN of the indicated mouse groups (*n =* 8 per group). **D**. Representative anti-TH immunohistochemistry images of the striatum coronal sections from saline or MPTP injection mouse groups administrated with either PBS or metformin water following the experimental plan shown in panel A. **E**. Quantification of TH fiber optical densities in panel B determined by ImageJ densitometry analysis (*n* = 8 per group). **F**. Pole test revealing behavioral benefit of metformin in MPTP intoxication model. (*n* = 7 per group).

Several studies have shown that PGC-1 expression and mitochondrial biogenesis are associated with the pathophysiology of dopaminergic neurons in PD [12, 14, 15, 17]. Since PGC-1α has been shown to play a neuroprotective role in several animal models of PD [12, 14, 17], we next tested whether metformin treatment and PGC-1α upregulation could be beneficial in the MPTP mouse model (Figure 5A). Unbiased stereological counting of MPTP-injected mice demonstrated an almost 40% loss of dopaminergic neurons in the SN pars compacta (Figures 5B and 5C). Conversely, treatment with metformin prevented MPTP-induced dopaminergic neurodegeneration, with a 20% loss of dopamine neurons (Figures 5B and 5C). Consistent with dopamine neuronal protection in the SN, metformin prevented severe loss of dopamine neuron fiber density from MPTP intoxication in the striatum as determined by TH immunohistochemistry followed by optical density measurement (Figures 5D and 5E). Furthermore, we applied pole test to measure dopamine-sensitive behavioral performance, demonstrating metformin not only protected dopaminergic neuronal death but improved motor behavior in MPTP mouse model (Figure 5F). This result suggests that metformin gives dopamine neurons the ability to resist the effects of the mitochondrial toxin MPTP.

### Metformin neuroprotective effect is dependent on PGC-1α induction

**Figure 6:**
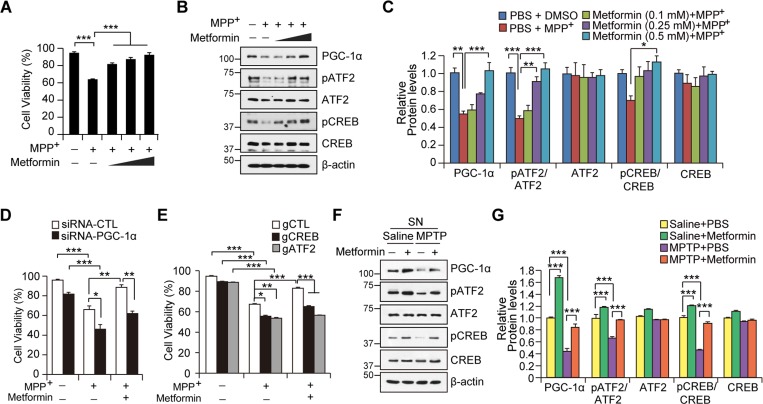
Metformin-mediated cell protection is dependent on PGC-1α induction **A**. Cell viability assessment in SH-SY5Y cells treated with the combination of 150 μM MPP+ and 0.1, 0.25, and 0.5 mM metformin for 48hr monitored by trypan blue exclusion assay (*n* = 3 independent experiments). **B**. Western blot of PGC-1α, CREB, and ATF2 in SH-SY5Y cells treated with the combination and doses of 150 μM MPP+ and 0.1, 0.25, and 0.5 mM metformin for 48hr. Phosphorylation of CREB and ATF2 was monitored by Western blot using the indicated antibodies. **C**. Quantification of PGC-1α and relative phosphorylation of CREB and ATF2 in SH-SY5Y cells treated with 150 μM MPP+ and/or metformin. Phospho-CREB and phospho-ATF2 band intensities were normalized to total CREB and ATF2, respectively (*n* = 3 per group). **D**. Cell viability assessment in SH-SY5Y cells with PGC-1α knockdown by transient siRNA transfection followed by treatment with 150 μM MPP+ or MPP+ and 0.5 mM metformin (*n* = 3 per group). Cell viability was monitored by trypan blue exclusion assay. **E**. Cell viability assessment in SH-SY5Y cells transfected with control guide RNA (gCTL), gCREB, or gATF2 followed by treatment with 150 μM MPP+ or MPP+ and 0.5 mM metformin (*n* = 3 per group). Cell viability was monitored by trypan blue exclusion assay. **F**. Expression of PGC-1α, CREB phosphorylation, and ATF2 phosphorylation in the SN of mice treated with metformin or MPTP or both as determined by Western blot with the indicated antibodies. **G**. Quantification of PGC-1α and relative phosphorylation of CREB and ATF2 in the SN of MPTP mice treated with either PBS or metformin. Phospho-CREB and phospho-ATF2 band intensities were normalized to total CREB and ATF2, respectively (*n* = 3 per group).

We next determined the role of PGC-1 in metformin-mediated cell survival. A trypan blue exclusion assay was used to assess SH-SY5Y cell viability in response to the mitochondrial toxin, 1-methyl-4-phenylpyridinium (MPP^+^). MPP^+^-induced cell toxicity in SH-SY5Y cells was rescued by metformin pretreatment in a dose-dependent manner (Figure 6A). Notably, we observed a marked reduction of phosphorylated ATF2 and CREB as well as PGC-1α repression by MPP^+^ treatment. On the other hand, pretreatment with metformin (0.5 mM) rendered SH-SY5Y able to maintain PGC-1α expression at basal levels even when challenged with MPP^+^ (Figure 6B). This result correlated with preservation of ATF2 and CREB phosphorylation by metformin pretreatment in MPP^+^-treated SH-SY5Y cells (Figures 6B and 6C). SH-SY5Y cell protection by metformin was dependent on PGC-1α expression, because metformin-mediated cell survival was largely abolished when PGC-1α was knocked down by siRNA transient transfection (Figure 6D, Supplementary Figures 4A and 4B). Consistent with the role of CREB and ATF2 in PGC-1α induction by metformin, gRNA-mediated deletion of CREB and ATF2 abolished the cell protective effects of metformin (Figure 6E). Further, physiological expression of PGC-1α, CREB, and ATF2 appeared to be critical for cell survival against mitochondrial stress, because SH-SY5Y cells with knockdown or deletion of these genes were more vulnerable to MPP^+^ toxicity compared to cells transfected with control DNA (Figures 6D and 6E)

Western blot analysis of the SN of MPTP mouse models revealed that metformin increased the levels of ATF2 and CREB phosphorylation as well as PGC-1 expression as compared to PBS-injected mice (Figures 6F and 6G), which may potentially contribute to the protective effects of metformin in dopaminergic neurons (Figures 5B-5F). Subsequent MPTP intoxication led to relative repression of PGC-1α expression in the SN of both mouse groups treated with either vehicle or metformin (Figures 6F and 6G). Significantly, the SN of metformin-treated mice demonstrated maintenance of PGC-1α expression and phosphorylation of ATF2 and CREB, the levels of which were equivalent to those in the control group (no MPTP + vehicle treatment). Overall, these *in vitro* and *in vivo* results suggest that PGC-1α induction by metformin plays a critical role in the survival of dopaminergic neurons in the presence of mitochondrial toxins and oxidative stress.

## DISCUSSION

### Global proteomic characterization of metformin in the substantia nigra

Metformin is an antidiabetic drug that has effects in several tissues important to metabolic homeostasis, including liver, skeletal muscle, adipose, and brain tissue. Considering its diverse physiological functions, understanding the metformin-induced proteomic changes in specific organs could provide insight into the molecular mechanisms of metformin's beneficial effects. Metformin treatment of animal models of nonalcoholic fatty liver disease has revealed differential expression of mitochondrial proteins, especially those related to metabolism, oxidative stress, and respiration [34]. Furthermore, mitochondrial subproteome analysis of mouse brains [35] has identified differential expression of proteins involved in metabolic processes, apoptosis, and mitochondria structure. Whole brain phosphoproteomic assessment has also suggested that mitochondrial proteins and proteins functioning in metabolism are altered [36]. The results of the present study provide additional insight into the effects of metformin on specific brain regions implicated in PD. Using unbiased proteomic assessment, we identified metabolic pathways and mitochondrial proteins as the main targets of metformin, as well as the global protein network altered by metformin that contributes to its neuroprotective functions in a PD mouse model.

### Brain region-specific effects of metformin

Metformin has been shown to induce PGC-1 expression in liver and skeletal muscle through poorly characterized molecular pathways [30, 37]. On the other hand, the ability of metformin to stimulate PGC-1α expression has not been examined in the mouse brain. In this study, we evaluate the induction of PGC-1α by metformin in the rodent brain and obtained interesting findings regarding the region specific effects of metformin. Specifically, metformin induced the expression of PGC-1α and mitochondrial proteins in the anatomical nigrostriatal pathway. To understand underlying mechanisms of this region-specific effect of metformin on PGC-1α expression will require further extensive studies. However, as predicted by the critical role of PGC-1α in cell function, *PGC-1α* transcription is tightly regulated by multiple transcription factors and their binding to distinct regions of the *PGC-1α* promoter. Indeed, differences in the basal regulation of the PGC-1α promoter in different regions of the brain might contribute to the differential responses seen with metformin. It is also likely that pharmacokinetic profile of metformin distribution in brain subregions have contributed to region specific effect of metformin in the CNS. Supporting this notion, we observed two fold higher amounts of metformin in CTX region as compared to SN region from mice with oral metformin administration. However, although PGC-1α is differentially induced by metformin in a brain-region selective manner, it should be noted that metformin's neuroprotective function can be mediated by signaling pathways other than PGC-1α in different regions of the brain.

### Molecular mechanisms of PGC-1α induction by metformin in mouse brains

As a master regulator of mitochondrial biogenesis, PGC-1α is involved in many biological processes in different organs. Specifically, PGC-1α is associated with neuroprotection in the brain, insulin resistance in the liver, and glucose sensing in skeletal muscle [19, 20]. In the brain, PGC-1α expression is critically relevant to dopaminergic neuronal survival [14, 15]. In PD brains, PGC-1α is repressed in sporadic PD patient brains as well as brains of Parkin inactivation [14]. Accumulation of the Parkin substrate PARIS has been shown to play a role in the repression of PGC-1α by binding to the insulin response sequence of the PGC-1α promoter [14]. Since PGC-1α repression and mitochondrial biogenesis defects are prevalent in many PD cases, and it has been shown that restoring PGC-1α expression is neuroprotective in PD models [14], development of chemical agents that maintain PGC-1α expression might represent a promising therapeutic target. Indeed, metformin has been already been shown to be neuroprotective in several neurodegenerative mouse models [24, 25, 38].

In the present study, we found that both the CRE/ATF2 motif in the *PGC-1α* promoter and ATF2/CREB activation by metformin were required for metformin-mediated induction of PGC-1α expression in SH-SY5Y cells and the mouse SN. It is not yet clear how metformin leads to activation of these transcription factors, but it is likely that the mild stress induced by metformin in the mitochondria contributes to some extent to eliciting signal cascade activation and subsequent phosphorylation of these transcription factors, which are known to function as stress sensors [32, 33]. Supporting this notion, we observed a slight increase in the levels of hydrogen peroxide in the presence of metformin. Furthermore, low concentrations of hydrogen peroxide led to PGC-1α induction and accompanying phosphorylation of CREB and ATF2. This area will require further study to explore the molecular mechanisms of nigrostriatal region-specific regulation of the ATF2/CREB-PGC-1α pathway by metformin.

### Dopaminergic neuroprotective effect of metformin determined using an MPTP mouse model and unbiased stereology

The neuroprotective functions of metformin remain controversial in PD mouse models. One group showed that induction of neurotrophic factors by metformin correlates with dopamine cell survival in MPTP mouse models *in vivo* [38]. Conversely, another PD research team, employing more extensive controls and experimental groups, showed that activation of 5′ AMP-activated protein kinase (AMPK) by metformin exacerbates dopaminergic cell loss in a 6-OHDA-induced PD mouse model [28]. In the present study, we employed unbiased stereological counting of dopaminergic neurons in the SN pars compacta to demonstrate that metformin pretreatment facilitates dopaminergic neuroprotection against MPTP intoxication. Since unbiased stereological counting is the gold standard in assessing dopamine neuron survival, our assessment of dopamine neurons confirmed the neuroprotective effects of metformin in the MPTP mouse model.

### PGC-1α induction is critical in metformin-mediated neuroprotective effects

As mentioned previously, PGC-1α repression is commonly observed in PD brains. Consistent with the role of PGC-1α in dopamine cell survival, siRNA knockdown of PGC-1α followed by trypan blue cell death experiments revealed the critical role of PGC-1α induction in mediating metformin's neuroprotective effects. However, we could not exclude the contribution of other signaling pathways to the neuroprotective effect of metformin. For example, AMPK is a well-known target of metformin and has been shown to be activated by metformin in various tissues. One report pointed out the adverse effects of AMPK activation by metformin in 6-OHDA-induced PD mouse models [28]. 6-OHDA itself also leads to AMPK activation, which correlates with dopaminergic cell loss. Although AMPK activation by metformin is potentially neuroprotective in drosophila PD animal models [29], its actual contribution to dopaminergic cell survival should be confirmed in future studies. Furthermore, a recent phosphoproteomic study indicated that metformin actually reduces AMPK phosphorylation in mouse brains, while AMPK phosphorylation is robustly enhanced in skeletal muscle [36]. Consistently, we failed to observe any changes in AMPK phosphorylation in SH-SY5Y neuroblastoma cells following treatment with 0.5 mM metformin in our study while other group observed AMPK phosphorylation by metformin at much higher concentration (2.5 mM) [39]. It appears metformin influences diverse signaling pathways depending on its concentration and cell types. Collectively, although AMPK is the well-studied indirect target of metformin, it is not certain whether metformin activates AMPK signaling in dopamine neurons or if such regulation is neuroprotective.

It is interesting that metformin induces PGC-1α expression via a CRE motif. PGC-1α repression in sporadic PD appears to be mediated by PARIS binding to insulin response sequence (IRS) motifs [14]. Thus, metformin could counteract the PGC-1α repressive effect of PARIS accumulation or PD conditions. Supporting this possibility, metformin treatment indeed elevates PGC-1α expression even in PD patients-derived adipocytes as well as MPTP mouse brains where Parkin inactivation and PARIS accumulation has been reported [39, 40]. Taken together, these findings suggest broad potential applications of metformin in the prevention of dopamine cell loss in PD cases involving PGC-1α dysfunction.

## MATERIALS AND METHODS

### Antibodies

The primary antibodies used in this study consisted of a mitochondrial marker antibody sampler kit (cat # 8674: rabbit antibody to SDHA (cat# 11998, 1:1,000, Cell Signaling), rabbit antibody to PDHA (cat# 3205, 1:1,000, Cell Signaling), rabbit antibody to VDAC (cat# 4661, 1:1,000, Cell Signaling), rabbit antibody to HSP60 (cat# 12165, 1:1,000, Cell Signaling), rabbit antibody to PHB1 (cat# 2426, 1:1,000, Cell Signaling), rabbit antibody to SDHA (cat# 11998, 1:1,000, Cell Signaling), rabbit antibody to COXIV (cat# 4850, 1:1,000, Cell Signaling)), rabbit antibody to tyrosine hydroxylase (NB300-109, 1:2,000, Novus Biologicals), mouse antibody to PGC-1α (cat# ST1202, 1:1,000, Calbiochem), rabbit antibody to CREB (cat# 9197, 1:1,000, Cell Signaling), rabbit antibody to phosphoCREB (cat# 9198, 1:1,000, Cell Signaling), mouse antibody to phospho-ATF2 (cat# sc-52941, 1:1,000, Santa Cruz), rabbit antibody to ATF2 (cat# sc-187, 1:1,000, Santa Cruz), mouse antibody to PARIS (cat#MABN476, 1:3000, Millipore) and an HRP-conjugated mouse antibody to b-actin (AC15, Sigma-Aldrich). The secondary antibodies used in this study were as follows: HRP-conjugated sheep antibody to mouse IgG (cat# RPN4301, 1:5,000, GE Healthcare), HRP-conjugated donkey antibody to rabbit IgG (cat# RPN4101, 1:5,000, GE Healthcare), and biotin-conjugated goat antibody to rabbit IgG (cat# BA-1000, 1:1,000, Vector Laboratories)

### Plasmids

The lentiCRISPR-gRNA to human *ATF2* or *CREB* was constructed by ligation of gRNA oligos into the BsmBI restriction site of the pLenti-CRISPR-v2 plasmid (Addgene #52961). The pCDNA3.1 myc/his PGC-1α construct was purchased from Addgene (#1026). The luciferase vector constructs, pGL3-PGC-1α promoter-Luciferase and CRE deletion mutant luciferase, were generously given by Dr. Bruce M. Spiegelman. Oligo sequences for generating gRNAs targeting each gene were as follows. ATF2: F- CACCGTCATCACTGGTAGTAGACTC, R- AAACGAGTCTACTACCAGTGATGAC; CREB: F-CACCGCTAATGTGGCAATCTGTGGC, R- AAACGCCACAGATTGCCACATTAGC. pGL3-*PGC1a-Luc* and its promoter mutant constructs have been described previously [14]. Construct integrity was verified by sequencing. Small interfering RNA to *PGC-1α* and scramble siRNA controls were purchased from Santa Cruz (*PGC-1* siRNA: sc-38884, control scramble siRNA: sc-93314).

### Cell culture and transfection

Human neuroblastoma SH-SY5Y cells (ATCC, Manassas, VA) were grown in DMEM containing 10% FBS (vol/vol) and antibiotics in a humidified 5% CO2/95% air atmosphere at 37°C. Adipose derived mesenchymal stromal cells (hAD-MSCs) [41] were grown in Mesenchymal Stem cell Expansion medium (Millipore, Billerica, MA, USA), and the culture media was replaced every 3 days. X-tremeGENE HP transfection reagent (Roche) was used for transient transfections according to the manufacturer's instructions. Unless otherwise indicated, lysates were prepared 48 h after transfection. For the luciferase assay, SH-SY5Y cells were transiently transfected with pGL3-Basic, pGL3-*PGC-1α* promoter-Luciferase, CRE deletion mutant luciferase constructs for firefly Luciferase assay, and 10 ng pRL-TK vector (Promega) for Renilla luciferase control.

### Luciferase assay

Cells were harvested 48 h after transfection with corresponding DNA constructs, and lysates were assayed for firefly luciferases using the Dual Luciferase Reporter Assay System (Promega, Fitchburg, USA) with a Glomax 20/20 luminometer (Promega, Fitchburg, USA), according to the manufacturer's instructions. Firefly luciferase levels were normalized using the Renilla control.

### Real-time quantitative PCR

Total RNA was extracted with Trizol (cat# 15596-026, Invitrogen) followed by DNase I treatment to eliminate trace DNA contamination. cDNA was synthesized from total RNA (1.5 mg) using a First-strand cDNA synthesis kit (cat# 11904-018, Invitrogen). Relative quantities of mRNA expression were analyzed using real-time PCR (RotorgeneQ, Qiagen). SYBR green PCR reagent (cat# 204074, Qiagen) was used according to the manufacturer's instructions. The primer sequences for real-time amplification of genes were as follows:

For human genes,

*PGC-1α*: F- TCCTCACAGAGACACTAGACA, R-CTGGTGCCAGTAAGAGCTTCT; *SOD1*: F- AGGGCATCATCAATTTCGAGC, R-GCCCACCGTGTTTTCTGGA;

*SOD2*: F- TTGGCCAAGGGAGATGTTAC, R-AGTCACGTTTGATGGCTTCC;

*NRF1*: F- CTTACAAGGTGGGGGACAGA, R-GGTGACTGCGCTGTCTGATA;

*TFAM*: F-CCGAGGTGGTTTTCATCTGT, R- TCCGCCTATAAGCATCTTG;

*GPX1*: F-GCACCCTCTCTTCGCCTTC, R- TCAGGCTCGATGTCAATGGTC;

*CAT1*: F-CGCAGAAAGCTGATGTCCTGA, R-TCATGTGTGACCTCAAAGTAGC;

*UCP2*: F-GCATCGGCCTGTATGATTCT, R-TTGGTATCTCCGACCACCTC;

*ACTB*: F-CATCCGCAAAGACCTGTACG, R-CCTGCTTGCTGATCCACATC.

For mouse genes,

*PGC-1α*: F-AGCCGTGACCACTGACAACGAG, R-CTGCATGGTTCTGAGTGCTAAG;

*SOD1*: F-CCAGTGCAGGACCTCATTTT, R- TTGTTTCTCATGGACCACCA;

*SOD2*: F-CCGAGGAGAAGTACCACGAG, R- GCTTGATAGCCTCCAGCAAC;

*NRF1*: F-GTTGGTACAGGGGCAACAGT, R-TCGTCTGGATGGTCATTTCA;

*TFAM*: F-CCAAAAAGACCTCGTTCAGC, R-CTTCAGCCATCTGCTCTTCC;

*GPX1*: F-CCGTGCAATCAGTTCGGACA, R-TCACTTCGCACTTCTCAAACAAT;

*CAT1*: F-AGCGACCAGATGAAGCAGTG, R-TCCGCTCTCTGTCAAAGTGTG;

*UCP2*: F-ACTTTCCCTCTGGATACCGC, R-ACGGAGGCAAAGCTCATCTG;

*ACTB*: F-CCTCTATGCCAACACAGTGC, R-CCTGCTTGCTGATCCACATC.

### Animal experiments

All animal experiments were approved by the Sungkyunkwan University Ethical Committee in accordance with international guidelines. Male C57BL/6N background mice were obtained from Orient (Suwon, Korea) and maintained at 12-h dark/light cycles in air-controlled rooms with access to diet and water ad libitum. All efforts were made to minimize animal suffering and to reduce the number of animals used. For proteomics, two group of eight-week-old mice were used, consisting of a control (PBS) group and metformin-treated group (Sigma-Aldrich, St. Louis, #D150959) (drinking water, 200 mg/kg/day, 14 days, *n* = 3 per group). Three groups of eight-week-old mice were used for biochemical analysis, consisting of a control group and two groups of metformin treatment at two different doses (Mid: 200 mg/kg/day, High: 400 mg/kg/day, 14 days drinking water, *n* = 3 per group). For MPTP toxicity experiments, eight-week-old mice were divided into four groups: PBS-saline, metformin-saline (200 mg/kg/day), PBS-MPTP, and metformin-MPTP (*n* = 10 per group). Metformin was administered to mice in drinking water, and treatment began on day 1 of the experiment and continued for 2 weeks, followed by stereological assessment of dopamine neuron numbers. MPTP was delivered by intra-peritoneal injection performed 4 times at 2hr intervals (Sigma-Aldrich, St. Louis, USA; 15 mg/kg) on day 7. Mouse brains were prepared as described below.

### MPTP injections and stereological TH neuron and fiber density assessment

After scheduled treatments with metformin in MPTP intoxication models and control groups, animals were anesthetized with pentobarbital (50 mg/kg, intraperitoneal injection) and perfused with PBS followed by 4% paraformaldehyde (wt/vol in PBS). Brains were post-fixed with 4% paraformaldehyde overnight and subsequently cryoprotected in 30% sucrose in PBS (wt/vol) overnight. Forty mm coronal sections were made throughout the brain including the substantia nigra and striatum, and every 4th section was utilized for analysis. For analysis of tyrosine hydroxylase (TH), sections were incubated with a 1:1000 dilution of rabbit polyclonal anti-TH (Novus) followed by sequential incubation with biotinylated goat anti-rabbit IgG and streptavidin-conjugated horseradish peroxidase (HRP) according to manufacturer's instructions (Vectastain ABC kit, Vector Laboratories, Burlingame, CA). 3,3-Diaminobenzidine (DAB, cat# D4293, Sigma) was used as substrate for HRP to visualize TH-positive cells. Total numbers of TH-positive neurons in the substantia nigra pars compacta were counted using the Optical Fractionator probe of Stereo Investigator software (MicroBrightfield, Williston, VT). Experimenters were blinded to the treatment during stereological counting. For TH fiber density assessment, images of striatum with TH immunohistochemistry were taken from each mouse brain section. Optical density of ventral striatum area (measured using image J) was first subtracted with adjacent background to normalize nonspecific staining. Normalized optical TH fiber density from each mouse striatum was used for statistical comparison.

### Pole test

Animals were acclimatized in the behavioral procedure room at least for 20 min. The pole is made up 58 cm metal rod with 10 mm diameter and wrapped with bandage gauze [40]. Briefly, the mice were placed on the top of the pole (7.5 cm from the top of the pole) facing the head-up. Total time taken to reach the base of the pole was recorded. Mice were evaluated in three sessions and total times and average were recorded. The maximum cutoff of time to stop the test and recording was 60 sec.

### Gel electrophoresis and Coomassie staining

Animals were perfused transcardially with phosphate-buffered saline (PBS) (pH 7.4) under pentobarbital anesthesia (50 mg/kg, intraperitoneal injection). Thirty milligrams of powderized substantia nigra or frontal cortex were then obtained and homogenized in RIPA buffer (Thermo Scientific). Concentrations of supernatants were determined by BCA assay. Total protein from mice in each group (PBS vs. metformin) was mixed with 1 ml of sample buffer containing 40 mM Tris, 5 M urea, 2 M thiourea, 4% CHAPS, 10 mM DTT, 1 mM EDTA, 0.5% IPG buffer 3-10 NL (GE Healthcare, USA), a protease/phosphatase inhibitor cocktail, and 1 mM PMSF. Samples were concentrated using Amicon™ centricon devices (Millipore, USA) and divided into aliquots containing appropriate amounts of protein for 1D separation. The samples were loaded on pre-cast gradient gels (4-20% Tris-glycine, GE Healthcare, USA). After running, the gels were incubated in fixation solution (50% methanol and 10% acetic acid) overnight, and proteins were visualized with colloidal Coomassie blue (Novex, San Diego, USA). Stained gels were scanned using an EPSON perfection V700.

### LC-MS/MS

Gel pieces were destained, reduced, alkylated, and digested with modified sequencing grade trypsin (Promega, USA). Peptide mixtures were resuspended in 0.1% TFA and injected in a Zorbox 300SB-C18 75 μm i.d. × 15 cm column (Agilent, Germany) via a trap column (Zorbox 300SB-C18 300 μm i.d. × 5 mm column, Agilent, Germany). Peptides were then separated in an acetonitrile gradient (buffer A - 0.1% formic acid; buffer B - 100% acetonitrile and 0.1% formic acid) at a flow rate of 200 nl/min with an UltiMate 3000 HPLC system (Dionex, USA) and applied on-line to an LTQ ion-trap mass spectrometer (Thermo Fisher, USA). The gradient started with an increase from 5% to 40% solution B over 110 min, followed by an increase to 80% B over 1 min, and then 80% B isocratic for 15 min. ESI ion source parameters were as follows: ion spray voltage 1.6 kV, capillary voltage 24 V, and capillary temperature 200°C. MS spectra were collected in full scan mode (350-1600 Da) followed by five MS/MS scans of the five most intense ions.

### Quantitative protein profiling, statistical analysis, and database searching

LC/MS data were analyzed with DeCyder MS software (version 2.0; GE Healthcare, Uppsala, Sweden). Peptide detection, background subtraction, and peptide quantitation were done on the full scan precursor mass spectra in fully automatic mode. Using PepDetect module, peptide peaks were detected with an average peak width of 1 min and matched with a mass accuracy of at least 0.6 Da. Data processing was manually inspected, and overlapping peaks were discarded. For quantitative comparison, the intensity distributions of all peptides detected in each sample were subject to normalization (no internal standards were added to the samples) and peptides were identified by importing Mascot™ 2.3 (Matrix Science, London, UK) search results to the PepMatch module. Proteins were identified by multiple peptides with a significant Mascot score (*p* < 0.05) as the following search parameters: enzyme: trypsin, missed cleavage sites allowed: 3, fixed modifications: carbamidomethyl, variable modifications: oxidation of methionine, precursor mass tolerance: 2 Da, and fragment mass tolerance: 1 Da. The threshold level for differentially expressed proteins was defined as a minimum 2-fold increase or decrease.

### Measurement of metformin levels in mouse brain

Sample preparation for brain tissues were performed as described with modifications [42]. Briefly, the brain samples were rinsed three times with PBS and homogenized in 100 mL of distilled water for 3 min at 4°C. Then, each sample was deproteinized with 1 mL of acetonitrile and 1.2 mL of methanol. After vortexing for 60s, the samples were centrifuged for 15 min at 16,000 X g. The supernatant was transferred into new tube and evaporated at 40°C under nitrogen stream. The resultant residues were dissolved with 30 mL of a mixture of acetonitrile:water (20:80, v/v). Ten microliters aliquots of the solutions were injected into an Agilent 1200 SL system (Agilent Technologies, Santa Clara, CA, USA) connected to an Q-Trap 3200 (AB Sciex, Concord, ON, Canada) equipped with ESI ion spray operated in the positive ion mode. The samples were separated in an RRHD Zorbax Eclipse Plus C18 analytical column (3.0×100 mm, 1.8 mm, Agilent Technology, Santa Clara, CA, USA) with a 15-min gradient. The mobile phases were used as 0.1% formic acid in water (A) and 0.1% formic acid in acetonitrile (B). After injection, the gradient was held for 2 min with 0% B, then increased from 0% B to 80% B for 13 min, and finally maintained for 10 min with 95% B. The autosampler temperature was kept at 10°C and the flow rate was set at 0.2 ml/min. The source parameters of the mass spectrometer during analysis were ionization voltage: 5,500 V, source temperature: 650°C. Metformin was quantified by following transition, m/z 130.1 ^®^ 60.1.

### In silico analysis of functional associations

The STRING 8.3 web server (http://string-db.org/) was used to generate functional association networks of the differentially expressed proteins identified between vehicle and metformin-treated SN or Cortex tissue.

### Preparation of tissues for immunoblot

Mouse brain tissues were harvested and homogenized in RIPA lysis buffer [50 mM Tris-HCl, pH 7.5, 150 mM NaCl, 2 mM EDTA, 1 % Triton X-100, 0.5 % sodium deoxycholate, 0.1 % SDS, Phosphate Inhibitor Cocktail I and II (Sigma), and Complete Protease Inhibitor Mixture (Roche)] using a Diax 900 homogenizer. After homogenization, samples were rotated at 4°C for 30 min to achieve complete lysis, after which RIPA-soluble and insoluble lysate was mixed with 2 X SDS sample buffer, followed by boiling for 10 min at 95°C. Protein levels were quantified using a BCA kit (Pierce) with BSA standards and analyzed by immunoblot. Immunoblotting was performed with the antibody of interest and visualized by chemiluminescence (Pierce). Densitometric analysis of immunoblot bands was performed using ImageJ (NIH, http://rsb.info.nih.gov/ij/).

### Cell viability analysis

SH-SY5Y cells were plated in 6-well plates at a seeding density of 0.5 x10^6^ cells per well. Following transient transfection with the indicated constructs, cells were grown in DMEM containing low serum (2.5 % FBS) with or without metformin for two more days. After treatment with MPP^+^ or DMSO vehicle, SH-SY5Y cells were trypsinized to yield single-cell suspensions that were washed twice with PBS before resuspension in serum-free DMEM. Resuspended cells were mixed with an equal volume of 0.4% trypan blue (wt/vol) and incubated for 2 min at room temperature. Live and dead cells were analyzed automatically (Eve™, nanoENTEK, Seoul, Korea).

### Statistics

Quantitative data are presented as the mean ± standard error of the mean (s.e.m.). Statistical significance was assessed either via an unpaired two-tailed Student's *t*-test for two-group comparisons or an ANOVA test with Tukey's HSD post hoc analysis for comparison of more than three groups. Assessments were considered significant at the level of *P* < 0.05.

### Data availability

Data related to this manuscript is available from the corresponding author upon reasonable request.

## SUPPLEMENTARY MATERIAL FIGURES AND TABLES





## References

[1] Lang AE, Lozano AM (1998). Parkinson's disease. Second of two parts. N Engl J Med.

[2] Lang AE, Lozano AM (1998). Parkinson's disease. First of two parts. N Engl J Med.

[3] Connolly BS, Lang AE (2014). Pharmacological treatment of Parkinson disease: a review. Jama.

[4] Bronstein JM, Tagliati M, Alterman RL, Lozano AM, Volkmann J, Stefani A, Horak FB, Okun MS, Foote KD, Krack P, Pahwa R, Henderson JM, Hariz MI (2011). Deep brain stimulation for Parkinson disease: an expert consensus and review of key issues. Arch Neurol.

[5] Moore DJ, West AB, Dawson VL, Dawson TM (2005). Molecular pathophysiology of Parkinson's disease. Annu Rev Neurosci.

[6] Shin JH, Dawson VL, Dawson TM (2009). SnapShot: pathogenesis of Parkinson's disease. Cell.

[7] Schober A (2004). Classic toxin-induced animal models of Parkinson's disease: 6-OHDA and MPTP. Cell Tissue Res.

[8] Cannon JR, Tapias V, Na HM, Honick AS, Drolet RE, Greenamyre JT (2009). A highly reproducible rotenone model of Parkinson's disease. Neurobiol Dis.

[9] Langston JW, Langston EB, Irwin I (1984). MPTP-induced parkinsonism in human and non-human primates—clinical and experimental aspects. Acta Neurol Scand Suppl.

[10] Haque ME, Thomas KJ, D'souza C, Callaghan S, Kitada T, Slack RS, Fraser P, Cookson MR, Tandon A, Park DS (2008). Cytoplasmic Pink1 activity protects neurons from dopaminergic neurotoxin MPTP. Proc Natl Acad Sci U S A.

[11] Yasuda T, Hayakawa H, Nihira T, Ren YR, Nakata Y, Nagai M, Hattori N, Miyake K, Takada M, Shimada T, Mizuno Y, Mochizuki H (2011). Parkin-mediated protection of dopaminergic neurons in a chronic MPTP-minipump mouse model of Parkinson disease. J Neuropathol Exp Neurol.

[12] Yu WH, Matsuoka Y, Sziraki I, Hashim A, Lafrancois J, Sershen H, Duff KE (2008). Increased dopaminergic neuron sensitivity to 1-methyl-4-phenyl-1,2,3,6-tetrahydropyridine (MPTP) in transgenic mice expressing mutant A53T alpha-synuclein. Neurochem Res.

[13] Karuppagounder SS, Xiong Y, Lee Y, Lawless MC, Kim D, Nordquist E, Martin I, Ge P, Brahmachari S, Jhaldiyal A, Kumar M, Andrabi SA, Dawson TM, Dawson VL (2016). LRRK2 G2019S transgenic mice display increased susceptibility to 1-methyl-4-phenyl-1,2,3,6-tetrahydropyridine (MPTP)-mediated neurotoxicity. J Chem Neuroanat.

[14] Shin JH, Ko HS, Kang H, Lee Y, Lee YI, Pletinkova O, Troconso JC, Dawson VL, Dawson TM (2011). PARIS (ZNF746) repression of PGC-1αlpha contributes to neurodegeneration in Parkinson's disease. Cell.

[15] Zheng B, Liao Z, Locascio JJ, Lesniak KA, Roderick SS, Watt ML, Eklund AC, Zhang-James Y, Kim PD, Hauser MA, Grunblatt E, Moran LB, Mandel SA (2010). PGC-1αlpha, a potential therapeutic target for early intervention in Parkinson's disease. Science translational medicine.

[16] Clark J, Reddy S, Zheng K, Betensky RA, Simon DK (2011). Association of PGC-1αlpha polymorphisms with age of onset and risk of Parkinson's disease. BMC Med Genet.

[17] Mudo G, Makela J, Di Liberto V, Tselykh TV, Olivieri M, Piepponen P, Eriksson O, Malkia A, Bonomo A, Kairisalo M, Aguirre JA, Korhonen L, Belluardo N, Lindholm D (2012). Transgenic expression and activation of PGC-1αlpha protect dopaminergic neurons in the MPTP mouse model of Parkinson's disease. Cell Mol Life Sci.

[18] Su X, Chu Y, Kordower JH, Li B, Cao H, Huang L, Nishida M, Song L, Wang D, Federoff HJ (2015). PGC-1αlpha Promoter Methylation in Parkinson's Disease. PLoS One.

[19] Puigserver P, Spiegelman BM (2003). Peroxisome proliferator-activated receptor-gamma coactivator 1 alpha (PGC-1 alpha): transcriptional coactivator and metabolic regulator. Endocr Rev.

[20] Fernandez-Marcos PJ, Auwerx J (2011). Regulation of PGC-1αlpha, a nodal regulator of mitochondrial biogenesis. Am J Clin Nutr.

[21] St-Pierre J, Drori S, Uldry M, Silvaggi JM, Rhee J, Jager S, Handschin C, Zheng K, Lin J, Yang W, Simon DK, Bachoo R, Spiegelman BM (2006). Suppression of reactive oxygen species and neurodegeneration by the PGC-1 transcriptional coactivators. Cell.

[22] Viollet B, Guigas B, Sanz Garcia N, Leclerc J, Foretz M, Andreelli F (2012). Cellular and molecular mechanisms of metformin: an overview. Clin Sci (Lond).

[23] Zhou G, Myers R, Li Y, Chen Y, Shen X, Fenyk-Melody J, Wu M, Ventre J, Doebber T, Fujii N, Musi N, Hirshman MF, Goodyear LJ, Moller DE (2001). Role of AMP-activated protein kinase in mechanism of metformin action. J Clin Invest.

[24] Cheng YY, Leu HB, Chen TJ, Chen CL, Kuo CH, Lee SD, Kao CL (2014). Metformin-inclusive therapy reduces the risk of stroke in patients with diabetes: a 4-year follow-up study. J Stroke Cerebrovasc Dis.

[25] Ma TC, Buescher JL, Oatis B, Funk JA, Nash AJ, Carrier RL, Hoyt KR (2007). Metformin therapy in a transgenic mouse model of Huntington's disease. Neurosci Lett.

[26] Yarchoan M, Arnold SE (2014). Repurposing diabetes drugs for brain insulin resistance in Alzheimer disease. Diabetes.

[27] Wahlqvist ML, Lee MS, Hsu CC, Chuang SY, Lee JT, Tsai HN (2012). Metformin-inclusive sulfonylurea therapy reduces the risk of Parkinson's disease occurring with Type 2 diabetes in a Taiwanese population cohort. Parkinsonism Relat Disord.

[28] Kim TW, Cho HM, Choi SY, Suguira Y, Hayasaka T, Setou M, Koh HC, Hwang EM, Park JY, Kang SJ, Kim HS, Kim H, Sun W (2013). (ADP-ribose) polymerase 1 and AMP-activated protein kinase mediate progressive dopaminergic neuronal degeneration in a mouse model of Parkinson's disease. Cell Death Dis.

[29] Ng CH, Guan MS, Koh C, Ouyang X, Yu F, Tan EK, O'Neill SP, Zhang X, Chung J, Lim KL (2012). AMP kinase activation mitigates dopaminergic dysfunction and mitochondrial abnormalities in Drosophila models of Parkinson's disease. J Neurosci.

[30] Suwa M, Egashira T, Nakano H, Sasaki H, Kumagai S (2006). Metformin increases the PGC-1αlpha protein and oxidative enzyme activities possibly via AMPK phosphorylation in skeletal muscle in vivo. J Appl Physiol (1985).

[31] Rolli M, Kotlyarov A, Sakamoto KM, Gaestel M, Neininger A (1999). Stress-induced stimulation of early growth response gene-1 by p38/stress-activated protein kinase 2 is mediated by a cAMP-responsive promoter element in a MAPKAP kinase 2-independent manner. J Biol Chem.

[32] Lopez-Bergami P, Lau E, Ronai Z (2010). Emerging roles of ATF2 and the dynamic AP1 network in cancer. Nat Rev Cancer.

[33] Ouwens DM, de Ruiter ND, van der Zon GC, Carter AP, Schouten J, van der Burgt C, Kooistra K, Bos JL, Maassen JA, van Dam H (2002). Growth factors can activate ATF2 via a two-step mechanism: phosphorylation of Thr71 through the Ras-MEK-ERK pathway and of Thr69 through RalGDS-Src-p38. Embo J.

[34] Stachowicz A, Suski M, Olszanecki R, Madej J, Okon K, Korbut R (2012). Proteomic analysis of liver mitochondria of apolipoprotein E knockout mice treated with metformin. J Proteomics.

[35] Suski M, Olszanecki R, Chmura L, Stachowicz A, Madej J, Okon K, Adamek D, Korbut R (2016). Influence of metformin on mitochondrial subproteome in the brain of apoE knockout mice. Eur J Pharmacol.

[36] Khang R, Park C, Shin JH (2014). The biguanide metformin alters phosphoproteomic profiling in mouse brain. Neurosci Lett.

[37] Aatsinki SM, Buler M, Salomaki H, Koulu M, Pavek P, Hakkola J (2014). Metformin induces PGC-1αlpha expression and selectively affects hepatic PGC-1αlpha functions. Br J Pharmacol.

[38] Patil SP, Jain PD, Ghumatkar PJ, Tambe R, Sathaye S (2014). Neuroprotective effect of metformin in MPTP-induced Parkinson's disease in mice. Neuroscience.

[39] Pérez-Revuelta BI, Hettich MM, Ciociaro A, Rotermund C, Kahle PJ, Krauss S, Di Monte DA (2014). Metformin lowers Ser-129 phosphorylated -synuclein levels via mTOR-dependent protein phosphatase 2A activation. Cell Death Dis.

[40] Ko HS, Lee Y, Shin JH, Karuppagounder SS, Gadad BS, Koleske AJ, Pletnikova O, Troncoso JC, Dawson VL, Dawson TM Phosphorylation by the c-Abl protein tyrosine kinase inhibits parkin's ubiquitination and protective function. Proc Natl Acad Sci U S A.

[41] Karuppagounder SS, Brahmachari S, Lee Y, Dawson VL, Dawson TM, Ko HS The c-Abl inhibitor, nilotinib, protects dopaminergic neurons in a preclinical animal model of Parkinson's disease. Sci Rep.

[42] Moon HE, Yoon SH, Hur YS, Park HW, Ha JY, Kim KH, Shim JH, Yoo SH, Son JH, Paek SL, Kim IK, Hwang JH, Kim DG (2013). Mitochondrial dysfunction of immortalized human adipose tissue-derived mesenchymal stromal cells from patients with Parkinson's disease. Exp Neurobiol.

[43] Łabuzek K, Suchy D, Gabryel B, Bielecka A, Liber S, Okopień B (2010). Quantification of metformin by the HPLC method in brain regions, cerebrospinal fluid and plasma of rats treated with lipopolysaccharide. Pharmacol Rep.

