# Supplementary material for: Activation of the ATF2/CREB-PGC-1α pathway by metformin leads to dopaminergic neuroprotection
Source: Oncotarget. 2017 May 24;8(30):48603–18. doi: 10.18632/oncotarget.18122 (PMC5564711; doi:10.18632/oncotarget.18122)
Supplement: Supplementary file 1 [file oncotarget-08-48603-s001.pdf]

Activation of the ATF2/CREB-PGC-1α pathway by metformin leads to dopaminergic neuroprotection

Supplementary Material

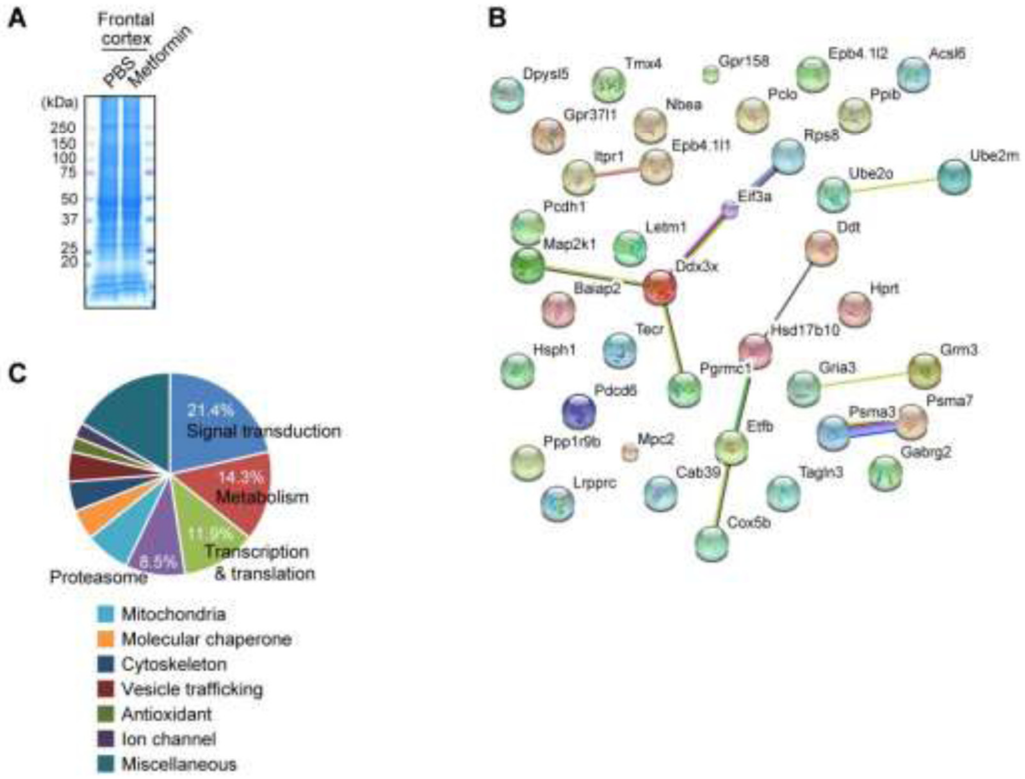

Supplementary Figure 1: Metformin induces global proteomic alterations in the mouse cortex
A. Representative 1DE image of cortex proteins of PBS- or metformin-administrated mice by Coommassie staining.
B. Proteins with differential expression between PBS and metformin treatment were analyzed by functional clustering. Line color annotation: green, neighborhood; red, gene fusion; blue, cooccurrence; black, coexpression; pink, experiments; light blue, databases; light green, text mining; light blue, homology.
C. Phi chart demonstrating biological functions enriched with multiple genes differentially expressed in the cortex of PBS- or metformin-administrated groups. The total number of genes with differential expression in each pathway was expressed as percentages.

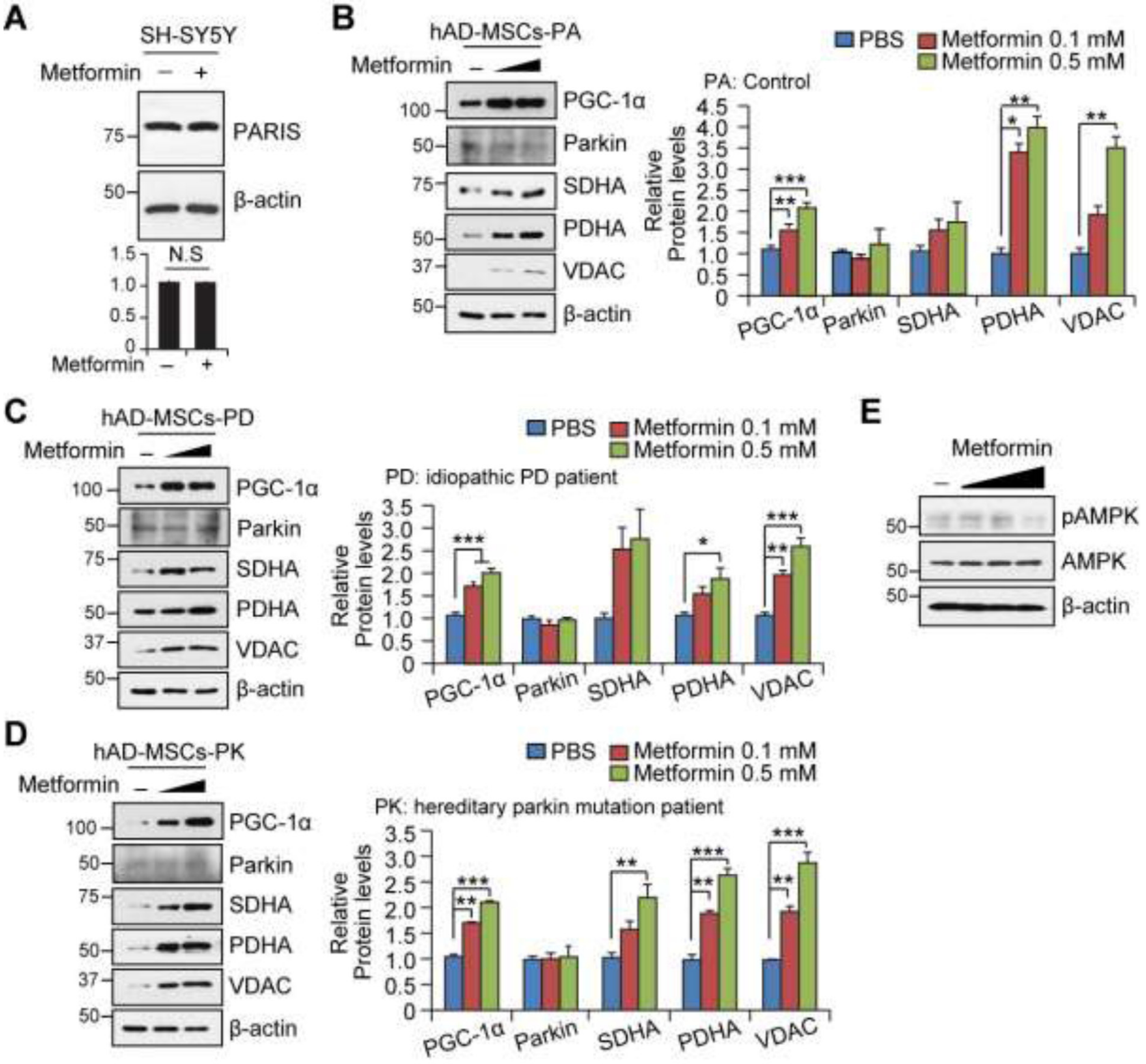

Supplementary Figure 2: Metformin induction of PGC-1a is independent of PARIS and parkin expression

A. PARIS expression in SH-SY5Y cells that were treated with metformin for 48hr determined by western blot using anti-PARIS antibody. Relative PARIS levels were normalized to β-actin and expressed as a bar graph (n = 3 independent experiments).

B~D. Representative western blots for PGC-1a, SDHA, PDHA, and VDAC in hAD-MSC cell lines derived from control (PA), idiopathic PD patient (PD), and patient with *Pakin* mutation (PK) that were treated with the indicated dose of metformin (left panels) for 48hr. Relative levels of each protein indicated were normalized to β-actin and compared to PBS treatment control set (n = 3 per group).

E. Total AMPK and phosphorylated AMPK (pAMPK) in SH-SY5Y cells treated with increasing dose of metformin for 48hr, determined by western bot using the indicated antibodies.

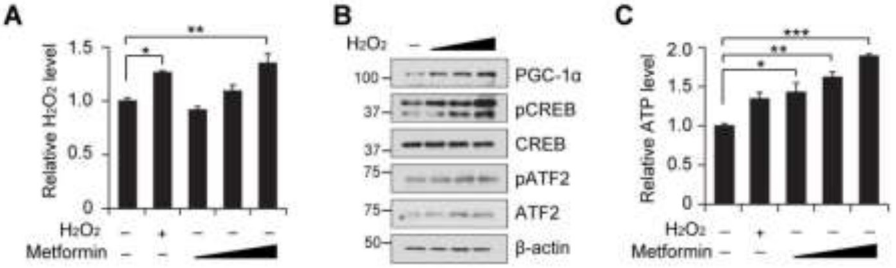

Supplementary Figure 3: Mild oxidative stress induced by metformin results in the increase of PGC-1α and ATP synthesis

A. Assessment of hydrogen peroxide levels as index of cellular oxidative stress in SH-SY5Y cells treated with metformin concentration (0.1, 0.25, 0.5 mM) or hydrogen peroxide (10 μM) for 48hr (n = 3 independent experiments).

B. Representative western blots of the indicated proteins in SH-SY5Y cells treated with the increasing low dose of hydrogen peroxide (5, 10, 50 μM) for 48hr.

C. Assessment of ATP levels in SH-SY5Y cells treated for 48hr with metformin (0.1, 0.25, 0.5 mM) or hydrogen peroxide (10 μM), (n = 3 independent experiments).

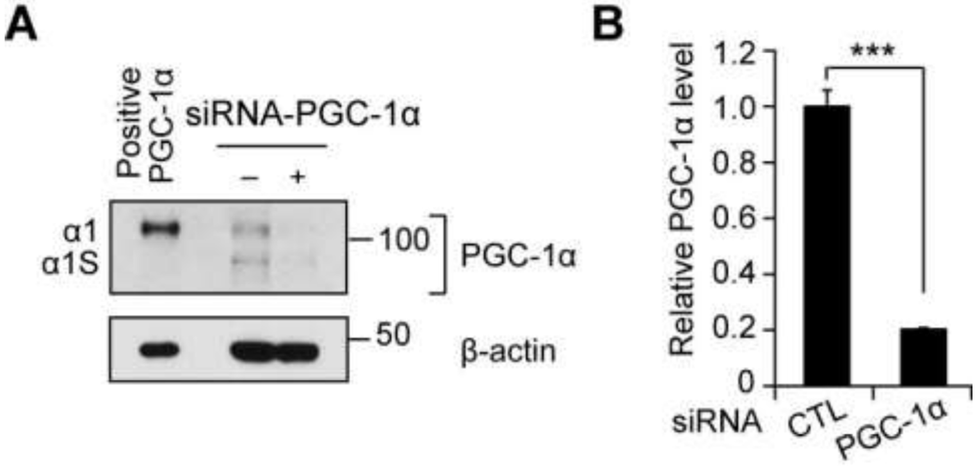

Supplementary Figure 4: Efficient knockdown of PGC-1α by siRNA

A. PGC-1α knockdown by transient transfection of siRNA to SH-SY5Y cells determined by western blot using anti-PGC-1α antibody. Scrambled siRNA transfection was used as control. **B**. Relative levels of PGC-1α protein (~120 kDa species assigned as PGC-1α1) were normalized to β-actin (n = 3 per group).

For Supplementary Tables see in Supplementary Files.
