# Supplementary material for: Activation of the ATF2/CREB-PGC-1α pathway by metformin leads to dopaminergic neuroprotection
Source: Oncotarget. 2017 May 24;8(30):48603–18. doi: 10.18632/oncotarget.18122 (PMC5564711; doi:10.18632/oncotarget.18122)
Supplement: Supplementary file 2 [file oncotarget-08-48603-s002.docx]

**Supplementary Information**

**Activation of ATF2/CREB-PGC-1α pathway by metformin leads to dopaminergic neuroprotection**

Hojin Kang, Rin Khang, Sangwoo Ham, Ga Ram Jung, Hyojung Kim, Minkyung Jo, Byeong Dae Lee, Yun Il Lee, Areum Jo, ChiHu Park, Hyein Kim, Jeongkon Seo, Sun Ha Paek, Yun-Song Lee, Jeong-Yun Choi, Yunjong Lee, and Joo-Ho Shin

**Supplementary Table 1. Proteins altered in the SN of metformin-administrated mice**

| **Acc. #** | **Protein name** | **#** | **Representative**  **Peptide Sequence** | **Intensity** | | **Av. Diff.**  **(^2^log)** |
| --- | --- | --- | --- | --- | --- | --- |
|  |  |  |  | **PBS** | **Met** |  |
| Metabolic enzymes | |  |  |  |  |  |
| P16330 | 2',3'-cyclic-nucleotide 3'-phosphodiesterase | 2 | RPPGVLHCTTK | 14.98 | 16.37 | -1.39 |
| Q8BGQ7 | Alanine--tRNA ligase, cytoplasmic | 3 | VGAEDADGIDMAYR | 15.50 | 16.77 | -1.26 |
| B0QZL1 | Alpha-enolase (Eno1) | 2 | IDKLMIEMDGTENK | 16.37 | 18.84 | -2.47 |
| P00920 | Carbonic anhydrase 2 | 3 | SIVNNGHSFNVEFDDSQDNAVLK | 17.68 | 19.38 | -1.70 |
| D3Z6E4 | Enolase (Eno2) | 2 | LAQENGWGVMVSHR | 18.26 | 19.56 | -1.30 |
| A6ZI44 | Fructose-bisphosphate aldolase | 4 | KELSDIAHR | 16.88 | 17.88 | -1.01 |
| P05063 | Fructose-bisphosphate aldolase C | 2 | ALQASALNAWR | 20.68 | 21.70 | -1.01 |
| F6SAC3 | Glucose-6-phosphate isomerase | 4 | HFVALSTNTAK | 19.72 | 20.93 | -1.21 |
| P48320 | Glutamate decarboxylase 2 | 2 | HYDLSYDTGDK | 18.01 | 19.42 | -1.41 |
| D3YU05 | Glyceraldehyde-3-phosphate dehydrogenase | 3 | IVSNASCTTNCLAPLAK | 17.18 | 18.44 | -1.26 |
| Q8CI94 | Glycogen phosphorylase, brain form | 4 | MSVIEEGDCK | 14.38 | 16.00 | -1.62 |
| G3UVV4 | Hexokinase 1 | 5 | VVDEYSLNSGK | 15.45 | 16.76 | -1.31 |
| P00493 | Hypoxanthine-guanine phosphoribosyltransferase | 2 | NVLIVEDIIDTGK | 18.08 | 19.11 | -1.03 |
| Q9CPU0 | Lactoylglutathione lyase | 3 | RFEELGVK | 17.50 | 19.59 | -2.09 |
| Q9EQ20 | Methylmalonate-semialdehyde dehydrogenase [acylating], mitochondrial | 2 | VPGATMLLAK | 18.43 | 19.49 | -1.06 |
| E9PZF0 | Nucleoside diphosphate kinase | 6 | GLVGEIIK | 17.28 | 18.53 | -1.25 |
| P09041 | Phosphoglycerate kinase 2 | 3 | VLNNMEIGTSLYDEEGAK | 18.35 | 21.38 | -3.03 |
| E9PUM3 | Phosphorylase | 2 | GYNAQEYYDR | 14.96 | 16.29 | -1.33 |
| P27773 | Protein disulfide-isomerase A3 | 3 | FAHTNIESLVK | 17.93 | 19.28 | -1.35 |
| E9Q7L0 | Protein Ogdhl | 3 | KPLIVFTPK | 15.60 | 18.08 | -2.48 |
| P52480 | Pyruvate kinase isozymes M1/M2 | 4 | LNFSHGTHEYHAETIK | 18.80 | 19.89 | -1.09 |
| P40142 | Transketolase | 2 | ILATPPQEDAPSVDIANIR | 18.62 | 19.63 | -1.00 |
| H7BXC3 | Triosephosphate isomerase | 6 | VSHALAEGLGVIACIGEK | 19.47 | 20.76 | -1.29 |
| *P50518* | *V-type proton ATPase subunit E 1* | *2* | *HMMAFIEQEANEK* | *18.87* | *16.96* | *1.91* |
| *P46460* | *Vesicle-fusing ATPase* | *2* | *LLDYVPIGPR* | *19.04* | *17.23* | *2.72* |
|  |  |  |  |  |  |  |
| Mitochondrial proteins | |  |  |  |  |  |
| Q60597 | 2-oxoglutarate dehydrogenase, mitochondrial | 2 | FEEFLQR | 16.13 | 17.42 | -1.30 |
| P63038 | 60 kDa heat shock protein, mitochondrial | 2 | VGEVIVTK | 16.20 | 17.45 | -1.25 |
| Q99KI0 | Aconitate hydratase, mitochondrial | 4 | VAVPSTIHCDHLIEAQVGGEK | 17.00 | 18.24 | -1.24 |
| P47738 | Aldehyde dehydrogenase, mitochondrial | 2 | VVGNPFDSR | 17.66 | 18.83 | -1.17 |
| D3Z6F5 | ATP synthase subunit alpha | 6 | AVDSLVPIGR | 18.39 | 20.27 | -1.88 |
| Q9CQQ7 | ATP synthase subunit b, mitochondrial | 2 | SISVQQEK | 16.08 | 12.84 | 3.24 |
|  |  |  |  |  |  |  |
|  |  |  |  |  |  |  |
| Table 1 (continued) | |  |  |  |  |  |
| **Acc. #** | **Protein name** | **#** | **Representative**  **Peptide Sequence** | **Intensity** | | **Av. Diff.**  **(^2^log)** |
|  |  |  |  | **PBS** | **Met** |  |
| P56480 | ATP synthase subunit beta, mitochondrial | 3 | IGLFGGAGVGK | 16.92 | 18.71 | -1.78 |
| F6XVM5 | ATP synthase subunit O, mitochondrial (Fragment) | 2 | YATALYSAASK | 16.95 | 19.61 | -2.66 |
| Q9CZ13 | Cytochrome b-c1 complex subunit 1, mitochondrial | 2 | NALVSHLDGTTPVCEDIGR | 18.34 | 19.49 | -1.15 |
| Q9DB77 | Cytochrome b-c1 complex subunit 2, mitochondrial | 2 | GNNTTSLLSQSVAK | 18.81 | 20.36 | -1.55 |
| O08749 | Dihydrolipoyl dehydrogenase, mitochondrial | 3 | ALTGGIAHLFK | 18.99 | 20.28 | -1.29 |
| E0CXD1 | Dynamin-like 120 kDa protein, mitochondrial | 2 | HEIELR | 15.29 | 16.45 | -1.16 |
| Q9D172 | ES1 protein homolog, mitochondrial | 2 | NVLELTGK | 17.84 | 19.09 | -1.25 |
| P26443 | Glutamate dehydrogenase 1, mitochondrial | 3 | ALASLMTYK | 19.14 | 20.38 | -1.24 |
| P54071 | Isocitrate dehydrogenase [NADP], mitochondrial | 2 | TIEAEAAHGTVTR | 16.45 | 17.94 | -1.50 |
| F6R307 | Methylglutaconyl-CoA hydratase, mitochondrial (Fragment) | 2 | AIGMSLAK | 15.47 | 16.85 | -1.38 |
| E9Q800 | Mitochondrial inner membrane protein | 3 | TAMDNSEIAGEK | 11.83 | 15.42 | -3.59 |
| Q9ERS2 | NADH dehydrogenase [ubiquinone] 1 alpha subcomplex subunit 13 | 2 | ENLEEEAIIMK | 17.58 | 18.88 | -1.30 |
| D3YUM1 | NADH dehydrogenase [ubiquinone] flavoprotein 1, mitochondrial | 3 | LVEGCLVGGR | 19.38 | 21.16 | -1.78 |
| Q91VD9 | NADH-ubiquinone oxidoreductase 75 kDa subunit, mitochondrial | 3 | VMNILHR | 17.94 | 19.06 | -1.11 |
| Q8K2B3 | Succinate dehydrogenase [ubiquinone] flavoprotein subunit, mitochondrial | 2 | SMQNHAAVFR | 17.45 | 18.72 | -1.27 |
| Q8BWF0 | Succinate-semialdehyde dehydrogenase, mitochondrial | 4 | VGNGFEEGTTQGPLINEK | 17.79 | 19.47 | -1.68 |
|  |  |  |  |  |  |  |
| Cytoskeletal proteins | |  |  |  |  |  |
| B1ATY1 | Actin, cytoplasmic 2 | 3 | EITALAPSTMK | 18.97 | 20.79 | -1.82 |
| Q99JY9 | Actin-related protein 3 | 2 | NIVLSGGSTMFR | 17.71 | 19.22 | -1.52 |
| P35564 | Calnexin | 2 | KTDAPQPDVK | 9.67 | 15.42 | -5.75 |
| Q5SXR6 | Clathrin heavy chain 1 | 2 | HDVVFLITK | 15.22 | 16.64 | -1.42 |
| P12960 | Contactin-1 | 3 | GSDNHSPISK | 11.33 | 13.43 | -2.09 |
| Q9R0P5 | Destrin | 2 | HEYQANGPEDLNR | 15.70 | 16.81 | -1.11 |
| O08553 | Dihydropyrimidinase-related protein 2 | 12 | FQMPDQGMTSADDFFQGTK | 18.91 | 20.35 | -1.45 |
| D3YUS0 | Dihydropyrimidinase-related protein 3 (Fragment) | 3 | AITVASQTNCPLYVTK | 15.54 | 18.52 | -2.98 |
| F6TH70 | Dynamin-1 (Fragment) | 2 | RSPTSSPTPQR | 6.78 | 17.37 | -10.59 |
| E9Q589 | Neural cell adhesion molecule 1 | 2 | QDDGGSPIR | 12.34 | 14.58 | -2.24 |
|  |  |  |  |  |  |  |
|  |  |  |  |  |  |  |
| Table 1 (continued) | |  |  |  |  |  |
| **Acc. #** | **Protein name** | **#** | **Representative**  **Peptide Sequence** | **Intensity** | | **Av. Diff.**  **(^2^log)** |
|  |  |  |  | **PBS** | **Met** |  |
| Q62261 | Spectrin beta chain, non-erythrocytic 1 | 6 | DTGNIGQER | 10.08 | 15.74 | -5.67 |
| O88935 | Synapsin-1 | 5 | TYATAEPFIDAK | 19.23 | 20.69 | -1.46 |
| Q64332 | Synapsin-2 | 2 | MNQLLSR | 16.01 | 17.61 | -1.60 |
| P62761 | Visinin-like protein 1 | 6 | LAPEVMEDLVK | 18.42 | 20.46 | -2.04 |
| *P08551* | *Neurofilament light polypeptide* | *6* | *LAAEDATNEK* | *18.44* | *17.20* | *1.24* |
|  |  |  |  |  |  |  |
| Ubiquitin-related proteins | |  |  |  |  |  |
| Q9CWH6 | Proteasome subunit alpha type-7-like | 2 | ALLEVVQSGGK | 17.53 | 18.69 | -1.15 |
| Q9R0P9 | Ubiquitin carboxyl-terminal hydrolase isozyme L1 | 2 | FSAVALCK | 19.53 | 21.18 | -1.65 |
| P61089 | Ubiquitin-conjugating enzyme E2 N | 2 | TNEAQAIETAR | 14.18 | 17.82 | -3.64 |
| B7ZBY6 | Ubiquitin-conjugating enzyme E2 variant 1 (Fragment) | 2 | VNMSGVSSSNGVVDPR | 14.53 | 17.04 | -2.51 |
| A6X925 | Ubiquitin-conjugating enzyme E2 variant 2 | 2 | INMNGINNSSGMVDAR | 14.23 | 15.61 | -1.38 |
| Q02053 | Ubiquitin-like modifier-activating enzyme 1 | 2 | QMNPYIQVTSHQNR | 15.78 | 16.78 | -1.00 |
| P31254 | Ubiquitin-like modifier-activating enzyme 1 Y | 3 | LVVADTR | 16.21 | 17.35 | -1.14 |
|  |  |  |  |  |  |  |
| Transcription and translation | |  |  |  |  |  |
| P63276 | 40S ribosomal protein S17 | 2 | VCEEIAIIPSK | 16.56 | 18.06 | -1.50 |
| B1ARA3 | 60S ribosomal protein L26 (Fragment) | 3 | IMSSPLSK | 14.20 | 16.35 | -2.15 |
| P21279 | Guanine nucleotide-binding protein G(q) subunit alpha | 3 | ILLLGAGESGK | 18.48 | 20.33 | -1.85 |
| Q9R1Q8 | Transgelin-3 | 2 | QGQNVIGLQMGSNK | 17.35 | 18.56 | -1.21 |
|  |  |  |  |  |  |  |
| Molecular chaperones | |  |  |  |  |  |
| F6VW30 | 14-3-3 protein theta (Fragment) | 5 | DSTLIMQLLR | 17.07 | 18.75 | -1.68 |
| *P61982* | *14-3-3 protein gamma* | *3* | *NCSETQYESK* | *17.61* | *8.25* | *9.36* |
| *D3YXF4* | *14-3-3 protein zeta/delta (Fragment)* | *2* | *YDDMAACMK* | *16.62* | *7.21* | *9.41* |
|  |  |  |  |  |  |  |
| Antioxidants | |  |  |  |  |  |
| P19157 | Glutathione S-transferase P 1 | 2 | ALPGHLKPFETLLSQNQGGK | 18.07 | 19.22 | -1.16 |
| D3Z0Y2 | Peroxiredoxin-6 | 5 | VVFIFGPDK | 15.98 | 18.96 | -2.98 |
| P08228 | Superoxide dismutase [Cu-Zn] | 8 | DGVANVSIEDR | 14.58 | 17.14 | -2.57 |
|  |  |  |  |  |  |  |
| Signal transduction | |  |  |  |  |  |
| P62204 | Calmodulin | 5 | EAFSLFDKDGDGTITTK | 16.81 | 18.11 | -1.30 |
| Q08331 | Calretinin | 6 | SGYIEANELK | 18.94 | 20.47 | -1.54 |
| P26645 | Myristoylated alanine-rich C-kinase substrate | 3 | VNGDASPAAAEPGAK | 14.63 | 15.83 | -1.20 |
|  |  |  |  |  |  |  |
| Table 1 (continued) | |  |  |  |  |  |
| **Acc. #** | **Protein name** | **#** | **Representative**  **Peptide Sequence** | **Intensity** | | **Av. Diff.**  **(^2^log)** |
|  |  |  |  | **PBS** | **Met** |  |
| Ion channels | |  |  |  |  |  |
| Q9D164 | FXYD domain-containing ion transport regulator 6 | 2 | EKDPFYYDYQTLR | 15.42 | 17.06 | -1.64 |
| F2Z3Z1 | Hippocalcin-like protein 4 (Fragment) | 4 | MNQDGLTPQQR | 13.77 | 16.81 | -3.03 |
|  |  |  |  |  |  |  |
| Vesicle trafficking | |  |  |  |  |  |
| P61205 | ADP-ribosylation factor 3 | 2 | DAVLLVFANK | 18.14 | 19.57 | -1.43 |
| P84086 | Complexin-2 | 2 | MLGGEEEKDPDAQKK | 7.43 | 15.58 | -8.15 |
|  |  |  |  |  |  |  |
| Miscellaneous | |  |  |  |  |  |
| *O55042* | *Alpha-synuclein* | *2* | *EGVVHGVTTVAEK* | *17.54* | *14.62* | *2.91* |
| Q9QXV0 | ProSAAS | 2 | AEAQEAEDQQAR | 7.30 | 14.09 | -6.79 |
| J3QNR5 | Uncharacterized protein | 12 | NMMAACDPR | 15.69 | 18.73 | -3.04 |
|  |  |  |  |  |  |  |

Acc. #, Accession number; #, number of peptide identified; Av. Diff. (^2^Log), average difference, positive or negative value indicates downregulation or upregulation, respectively. The down-regulated proteins are in Italic.

**Supplementary Table 2. Proteins altered in the cortex of metformin-administrated mice**

| **Acc. #** | **Gene**  **Name** | **Protein name** | **Intensity** | | **Fold**  **change** | |
| --- | --- | --- | --- | --- | --- | --- |
|  |  |  | **PBS** | **Met** |  |  |
| **Signal transduction** | | | | | | |
| P22723 | Gabrg2 | Gamma-aminobutyric acid receptor subunit gamma-2 | 2 | 7 | 3.7 | ↑ |
| Q8C419 | Gpr158 | Probable G-protein coupled receptor 158 | 8 | 15 | 2 | ↑ |
| Q99JG2 | Gpr37l1 | Prosaposin receptor GPR37L1 | 5 | 10 | 2.1 | ↑ |
| B0QZW1 | Gria3 | Glutamate receptor 3 | 6 | 15 | 2.6 | ↑ |
| Q9QYS2 | Grm3 | Metabotropic glutamate receptor 3 | 10 | 23 | 2.4 | ↑ |
| *P11881* | *Itpr1* | *Inositol 1,4,5-trisphosphate receptor type 1* | *4* | *2* | *0.5* | ↓ |
| *P31938* | *Map2k1* | *Dual specificity mitogen-activated protein kinase kinase 1* | *6* | *3* | *0.5* | ↓ |
| *O55022* | *Pgrmc1* | *Membrane-associated progesterone receptor component 1* | *10* | *3* | *0.3* | ↓ |
| *Q6R891* | *Ppp1r9b* | *Neurabin-2* | *5* | *2* | *0.4* | ↓ |
| **Miscellaneous** | | | | | | |
| *B1AZ46* | *Baiap2* | *Brain-specific angiogenesis inhibitor 1-associated protein 2* | *12* | *6* | *0.5* | ↓ |
| *E9PV14* | *Epb41l1* | *Band 4.1-like protein 1* | *15* | *6* | *0.4* | ↓ |
| *O70318* | *Epb41l2* | *Band 4.1-like protein 2* | *8* | *4* | *0.5* | ↓ |
| *A0A087WP80* | *Lsamp* | *Limbic system-associated membrane protein* | *7* | *2* | *0.3* | ↓ |
| *F7BJK1* | *Pcdh1* | *Protocadherin 1* | *12* | *5* | *0.4* | ↓ |
| P12815 | Pdcd6 | Programmed cell death protein 6 | 2 | 4 | 2.1 | ↑ |
| G3UWE1 | Tecr | MCG11048, isoform CRA_c | 4 | 9 | 2.3 | ↑ |
| **Metabolic enzymes** | | | | | | |
| Q91WC3 | Acsl6 | Long-chain-fatty-acid--CoA ligase 6 | 8 | 19 | 2.5 | ↑ |
| O35215 | Ddt | D-dopachrome decarboxylase | 10 | 19 | 2 | ↑ |
| *Q9EQF6* | *Dpysl5* | *Dihydropyrimidinase-related protein 5* | *22* | *8* | *0.4* | ↓ |
| *P00493* | *Hprt1* | *Hypoxanthine-guanine phosphoribosyltransferase* | *14* | *7* | *0.5* | ↓ |
| *A2AFQ2* | *Hsd17b10* | *3-hydroxyacyl-CoA dehydrogenase type-2* | *4* | *2* | *0.5* | ↓ |
| *P24369* | *Ppib* | *Peptidyl-prolyl cis-trans isomerase B* | *18* | *9* | *0.5* | ↓ |
| **Transcription/translation** | | | | | | |
| Q62167 | Ddx3x | ATP-dependent RNA helicase DDX3X | 7 | 14 | 2.1 | ↑ |
| *P23116* | *Eif3a* | *Eukaryotic translation initiation factor 3 subunit A* | *6* | *3* | *0.5* | ↓ |
| Q9Z2I0 | Letm1 | LETM1 and EF-hand domain-containing protein 1, mitochondrial | 4 | 11 | 2.9 | ↑ |
| P62242 | Rps8 | 40S ribosomal protein S8 | 5 | 10 | 2.1 | ↑ |
| Q9R1Q8 | Tagln3 | Transgelin-3 | 4 | 12 | 3.1 | ↑ |
| **Proteasome/Ubiquitination** | |  |  |  |  |  |
| *O70435* | *Psma3* | *Proteasome subunit alpha type-3* | *6* | *3* | *0.5* | ↓ |
| *Q9Z2U0* | *Psma7* | *Proteasome subunit alpha type-7* | *5* | *2* | *0.4* | ↓ |
| P61082 | Ube2m | NEDD8-conjugating enzyme Ubc12 | 3 | 10 | 3.5 | ↑ |
| *Q6ZPJ3* | *Ube2o* | *E2 ubiquitin-conjugating enzyme UBE2O* | *5* | *2* | *0.4* | ↓ |
| **Mitochondria** |  |  |  |  |  |  |
| P19536 | Cox5b | Cytochrome c oxidase subunit 5B, mitochondrial | 2 | 7 | 3.7 | ↑ |
| *Q9DCW4* | *Etfb* | *Electron transfer flavoprotein subunit beta* | *5* | *2* | *0.4* | ↓ |
| Q9D023 | Mpc2 | Mitochondrial pyruvate carrier 2 | 5 | 15 | 3.1 | ↑ |
| Table 2 (continued) | | | | | | |
| **Acc. #** | **Gene**  **Name** | **Protein name** | **Intensity** | | **Fold**  **change** | |
|  |  |  | **PBS** | **Met** |  |  |
| **Cytoskeleton** |  |  |  |  |  |  |
| *A0A087WQE8* | *Kif1a* | *Kinesin-like protein KIF1A* | *7* | *2* | *0.3* | ↓ |
| A0A1D5RLK2 | Psd3 | PH and SEC7 domain-containing protein 3 | 3 | 6 | 2.1 | ↑ |
| **Molecular chaperone** | | | | | | |
| *E9Q0U7* | *Hsph1* | *Heat shock protein 105 kDa* | *11* | *4* | *0.4* | ↓ |
| *Q6PB66* | *Lrpprc* | *Leucine-rich PPR motif-containing protein, mitochondrial* | *32* | *13* | *0.4* | ↓ |
| **Vesicle trafficking** | | | | | | |
| Q9EPN1 | Nbea | Neurobeachin | 9 | 25 | 2.9 | ↑ |
| Q9QYX7 | Pclo | Protein piccolo | 9 | 17 | 2 | ↑ |
| **Antioxidant** | | | | | | |
| A2ARI0 | Tmx4 | Thioredoxin-related transmembrane protein 4 | 2 | 6 | 3.1 | ↑ |
| **Ion channel** | | | | | | |
| *Q06138* | *Cab39* | *Calcium-binding protein 39* | *7* | *3* | *0.4* | ↓ |

Acc. #, Accession number; #, Arrows (↑ and ↓) indicate upregulation or downregulation, respectively. The down-regulated proteins are in Italic.
